# DNA Methylation Is Crucial for 1-Methylcyclopropene Delaying Postharvest Ripening and Senescence of Tomato Fruit

**DOI:** 10.3390/ijms26010168

**Published:** 2024-12-28

**Authors:** Zhiqiang Wang, Jinmei Xie, Wenhui Duan, Zhengke Zhang, Lanhuan Meng, Lisha Zhu, Qing Wang, Hongmiao Song, Xiangbin Xu

**Affiliations:** 1 Key Laboratory of Food Nutrition and Functional Food of Hainan Province, School of Food Science and Engineering, Hainan University, Haikou 570228, China; zhiqiangwangn@163.com (Z.W.); xiejinmei@hainanu.edu.cn (J.X.); duanwhmn@163.com (W.D.); zhangzhengke@hainanu.edu.cn (Z.Z.); huanhuanaini1012@126.com (L.M.); 996454@hainanu.edu.cn (L.Z.); 2 Key Laboratory of the Vegetable Postharvest Treatment of Ministry of Agriculture, Beijing Key Laboratory of Fruits and Vegetable Storage and Processing, Institute of Agri-Food Processing and Nutrition (IAPN), Beijing Academy of Agriculture and Forestry Sciences, Beijing 100097, China; wangqing@nercv.org.cn

**Keywords:** DNA methylation, tomato, ripening, senescence, 1-methylcyclopropen

## Abstract

DNA methylation is an epigenetic modification process that can alter the functionality of a genome. It has been reported to be a key regulator of fruit ripening. In this study, the DNA methylation changes of CpG islands of ethylene signaling genes regulated by 1-methylcyclopropene (1-MCP) during ripening and senescence of tomato fruit were detected. The results showed that the 1-MCP treatment decreased the accumulation of lycopene, maintained the content of vitamin C, and delayed the ripening and senescence of tomato fruit. The quantitative real-time PCR and bisulfite sequencing analysis showed that 1-MCP treatment changed the expression and the DNA methylation level of CpG islands related to the ethylene signaling pathway genes, among which the DNA methylation change of *LeEIN3* was the most significant. Compared with the control, 1-MCP treatment increased the DNA methylation level of the CpG island of the *LeEIN3* gene, reduced the expression of *LeEIN3* in tomato fruit, and was involved in 1-MCP delaying the postharvest senescence of tomato fruit. The results indicated that DNA methylation changes of ethylene signaling genes were involved in ethylene synthesis and signal transduction and played an important role in the regulation of 1-methylcyclopropene, delaying postharvest ripening and senescence of tomato fruit.

## 1. Introduction

Fruit senescence, a developmentally programmed degeneration process, is the final step of fruit development. It leads to a short storage life and bad quality and often causes great economic loss. Inhibition of ethylene biosynthesis and signaling is an effective technology to delay postharvest senescence of climacteric fruit. 1-methylcyclopropene (1-MCP), a cyclic alkene, can bind the ethylene receptor and thus block ethylene signaling and prevent the ripening and senescence-related genes from activating [1,2]. Studies have shown that the fumigation of 1-MCP significantly extended the storage shelf life of fruit and vegetables, maintained the postharvest fruit quality, and delayed the ripening and senescence of climacteric fruit [2]. The treatment of 1-MCP significantly suppressed the anthracnose of mango fruit, delayed mango fruit ripening, delayed mango fruit softening, and inhibited ethylene production and the respiration rate [3]. 1-MCP is involved in delaying banana fruit ripening and senescence by inhibiting the expression of genes associated with the ethylene signaling pathway [4]. Treatment with 1-MCP postharvest can suppress the production of ethylene and the respiration rate, delay the development of surface color, and decrease the accumulation of lycopene, thereby delaying the tomato ripening and senescence [5]. Treatment with 1-MCP has been shown to decrease the expression levels of the *Ad-ERS1a*, *Ad-ETR2*, *Ad-ETR3,* and *Ad-ERFs* genes, thereby delaying the kiwifruit fruit ripening and senescence [6]. In addition, the application of 1-MCP to tomato fruit declined the transcription levels of *SlACO1*, *SlACO4*, *SlACS2,* and *SlACS4* [7] and inhibited the expression of the ethylene receptors *LeETRs* (*LeETR1*, *LeETR2*, *LeETR3*, *LeETR4*, and *LeETR6*) and *LeCTRs* (*LeCTR1*, *LeCTR2*, *LeCTR3*, and *LeCTR4*) [8]. It also blocked ethylene signaling and delayed fruit ripening and senescence.

DNA methylation is a chemical modification process that takes place under the catalytic effect of DNA methyltransferases (DNMTs). In this process, S-adenosylmethionine (SAM) acts as the methyl donor, and the methyl group is covalently attached to specific bases in the DNA chain [9,10]. This modification does not change the nucleotide sequence of the DNA but can affect gene expression and regulation [9,10]. DNA methylation is an epigenetic modification that is widely present in eukaryotic organisms. It plays an important role in biological processes, such as genomic imprinting and disease defense, X chromosome abnormality, transposon silencing, carcinogenesis, and senescence [11,12,13]. In plants, DNA methylation commonly occurs at cytosine sites in three sequences, such as CG, CHG, and CHH (in which A, C, or T) [14]; these three types of methylation have different maintenance mechanisms. Methylation at CG and CHG sites is maintained by Methyltransferase1 (MET1) and Chromomethylase3 (CMT3) [15], whereas methylation at CHH sites is maintained by Domains Rearranged Methyltransferases (DRM1 and DRM2) [16] and CMT2 methyltransferase [17], respectively. DNA methylation was closely related to fruit ripening and senescence [18].

DNA methylation influenced N6-methyladenosine (m6A) modification by regulating the expression of the m6A demethylase gene, which, in turn, regulates DNA methylation via m6A demethylase feedback, thereby regulating tomato fruit ripening [19]. In pears, *CAMTA2* is identified as a gene associated with highly differentially methylated regions and its overexpression in tomatoes has been verified to delay fruit ripening, suggesting that hypermethylated and differentially methylated regions play an important role in fruit ripening [20].

Heat treatment delayed the postharvest ripening of tomato fruit by inducing the changes in DNA methylation levels of the *SlACS10*, *LeCTR1*, *LeEIN3*, *LeERT10*, and *SlERF-A1* genes [21]. Melatonin treatment increased the expression level of *SlACS10*, *SlEIN3*, *SlERF-A1,* and *SlERT10* by regulating the decreases in DNA methylation levels of *SlACS10*, *SlEIN3*, *SlERF-A1,* and *SlERT10*, suggesting that DNA methylation plays an important role in the regulation of ethylene signal transmission and tomato fruit ripening [22]. *PpNAC1* functions as a positive regulator of fruit ripening, exhibiting increased transcript levels upon overexpression, which correlates with reductions in DNA methylation within the promoters of its ripening-related target genes, including *PpACS1, PpACO1, PpPME1, PpPL1, PpPG1, PpLIP1, PpFAD3-1*, and *PpAAT1*, during the ripening process [23]. In the promoter regions of *MdMYB10,* differential methylation occurred, which resulted in the transcription of anthocyanin structural genes and pigment accumulation during apple fruit ripening [24]. In melon fruit, *CTR1-like* and *ROS1* have been identified as crucial ripening regulators, with *CTR1-like* functioning as a negative regulator and *ROS1* as a DNA demethylase ortholog. Both loss-of-function mutants *CTR1-like* and *ROS1* exhibit earlier ethylene production compared to the wild-type. Notably, a *ROS1* knockout specifically causes DNA hypomethylation in the promoter regions related to the ethylene signaling pathway genes, such as *ACS1*, *ETR1*, and *ACO1*, and ripening-associated transcription factors, like *NAC-NOR*, *RIN*, and *CNR*, highlighting the significant role of significant role in melon fruit ripening [25]. The application of a DNA methylation inhibitor delayed orange fruit ripening [26]. During the ripening process of strawberry fruit, the expression of key RdMM (RNA-guided DNA methylation) genes, such as *DRM2* and *AGO4* genes, is downregulated, leading to a decrease in DNA methylation, indicating that DNA methylation plays an important role in the fruit ripening process [27]. At the ripening stage of tomato fruit, the levels of DNA methylation were high in *Cnr* (encoding the transcription factor colorless non-ripening) and *rin* (ripening-inhibitor) mutants, whilethe global methylation levels of the wild-type also gradually declined [18]. The DNA hypermethylation upstream of the *SlSPL-Cnr* promoter caused the *Cnr* mutant, which influenced the normal ripening of tomato fruit [28].

In this study, to further understand the epigenetic mechanism of 1-MCP in delaying fruit ripening and senescence, the DNA methylation profile changes of CpG islands of *SlACS10*, *LeCTR1*, *LeEIN3,* and *SlERF-A1* genes regulated by 1-MCP in tomato fruit were detected and analyzed.

## 2. Results

### 2.1. Effects of 1-MCP Treatment on Tomato Fruit Ripening, Senescence, and Related Physiological Indexes

**Figure 1:**
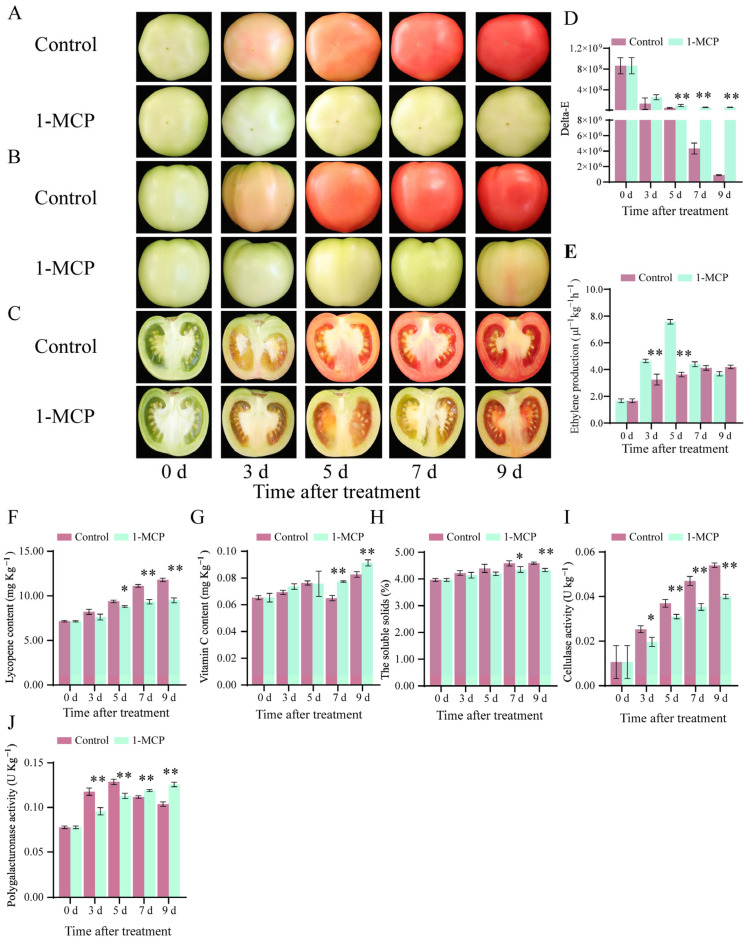
The phenotypic characterization of ripening and senescence changes of tomato fruit. (**A**) The top view of tomato fruit. (**B**) The lateral view of tomato fruit. (**C**) The longitudinal profile view of tomato fruit. (**D**) The pericarp color delta-E. (**E**) Ethylene production, (**F**) The lycopene content, vitamin C (**G**), the soluble solids (**H**), cellulase (**I**), and polygalacturonase activities (**J**) in tomato fruit. Asterisks indicate statistical differences in the values at *p* < 0.05 (*) or *p* < 0.01 (**).

Compared with the control, 1-MCP treatment delayed the ripening and senescence of tomato fruit (Figure 1). The color of 1-MCP treated fruit remained green from 0 to 9 d (Figure 1A,B) and the longitudinal profile of pulp tissue was pink from 3 to 9 d (Figure 1C), while the color of control fruit became red after 7 d of storage (Figure 1).

During storage from 0 to 9 d, the delta-E values of 1-MCP-treated tomato fruit showed a gradually decreasing trend, while the delta-E values of control tomato fruit showed a sharp decreasing trend (Figure 1D). Compared with the control, the peel of 1-MCP-treated fruit showed higher delta-E values (Figure 1D). The ethylene production of the control group gradually increased at 5 days, reached its peak at 5 days, and then decreased (Figure 1E). During storage from 0 to 9 d, the ethylene production gradually increased with 1-MCP treatment but was lower than that of the control group (Figure 1E).

During storage from 3 to 9 d, the content of lycopene increased by 24.50% in 1-MCP-treated tomato fruit and increased by 43.67% in control fruit (Figure 1F). Compared with the control fruit, the accumulation of lycopene was significantly reduced in fruits treated with 1-MCP (Figure 1F). Vitamin C is the main antioxidant molecule in tomato fruit, which protects the organism from free radicals. In the 1-MCP-treated fruit, the content of vitamin C remained relatively constant throughout storage. During storage from 3 to 9 d, the content of vitamin C increased by 18.84% in 1-MCP-treated tomato fruit and increased by 17.98% in control fruit (Figure 1G). Compared with the control fruit, the accumulation of vitamin C in the fruit treated with 1-MCP significantly increased (Figure 1G). The total soluble solids are one of the important indicators of the ripening and senescence of fruit. During the storage from 3 to 9 d, the total soluble solids increased by 4.61% in 1-MCP-treated tomato fruit and increased by 8.76% in control fruit (Figure 1H). Compared with the control fruit, there was no significant change in the total soluble solids in the fruit treated with 1-MCP (Figure 1H). The activities of cellulase and polygalacturonase are related to cell wall degradation. The decomposition of cell wall components and structure leads to the loss of firmness or softening of the fruit, which is closely related to the ripening and senescence of the fruit. From 3 to 9 d of storage, the cellulase activity in 1-MCP-treated tomato fruit increased by 104.62% and increased by 113.51% in control fruit (Figure 1I). Compared with the control fruit, there was no significant change in cellulase activity in the fruit treated with 1-MCP (Figure 1I). During storage from 0 to 5 d, the polygalacturonase activity of tomato fruit in the two groups exhibited an increasing trend during storage (Figure 1J). During storage from 0 to 5 d, compared with the control fruit, there was no significant change in polygalacturonase activity in the fruit treated with 1-MCP (Figure 1J). The polygalacturonase activity increased by 37.04% in 1-MCP-treated tomato fruit and increased by 43.37% in control fruit (Figure 1J). From 5 to 9 d of storage, the polygalacturonase activity increased by 13.23% in 1-MCP-treated fruit and decreased by 21.18% in control fruit (Figure 1J). During storage from 5 to 9 d, compared with the control fruit, there was a significant change in polygalacturonase activity in the fruit treated with 1-MCP (Figure 1J).

### 2.2. Effects of 1-MCP on Methylase and Demethylase Activities in Tomato Fruit

**Figure 2:**
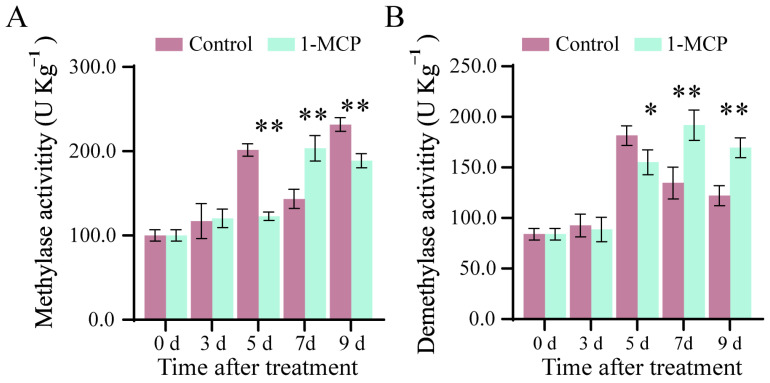
Effects of 1-MCP on the activities of methylase (**A**) and demethylase (**B**) in tomato fruit. Vertical bars represent standard deviations of the means. Asterisks indicate statistical differences in the values at *p* < 0.05 (*) or *p* < 0.01 (**).

During storage from 3 to 9 d, the methylase activity gradually increased with fruit ripening. After storage for 3 d, the activity of methylase in 1-MCP-treated fruit and control fruit was 120.36 and 117.22 U Kg^−1^, respectively (Figure 2A). After storage for 7 and 9 d, the methylase activities were 203.51 and 188.78 U Kg^−1^, and 143.45 and 231.71 U Kg^−1^ in 1-MCP-treated fruit and control fruit, respectively (Figure 2A). The demethylase activities in 1-MCP-treated tomato fruit were higher than that in control at the later stage of storage (Figure 2B). After storage for 7 and 9 d, the demethylase activities were 191.77 and 169.48 U Kg^−1^, and 134.63 and 122.03 U Kg^−1^ in 1-MCP-treated fruit and control fruit, respectively (Figure 2B).

### 2.3. Effects of 1-MCP on DNA Methylation and the Genes Expression Levels

To understand the roles of DNA methylation on the ripening and senescence of tomato fruit, the DNA methylation changes of the CpG islands of *SlACS10*, *LeCTR1*, *LeEIN3,* and *SlERF-A1* and their expression were analyzed. The methylation sites of the CpG islands of *SlACS10*, *LeCTR1*, *LeEIN3,* and *SlERF-A1* were located between the promoter and the first exon region (−45 to +309 bp), in the first exon region (+130 to +323 bp), the exon region (+92 to +286 bp), and the exon region of downstream (+334 to +677 bp), respectively (Supplementary S1).

**Figure 3:**
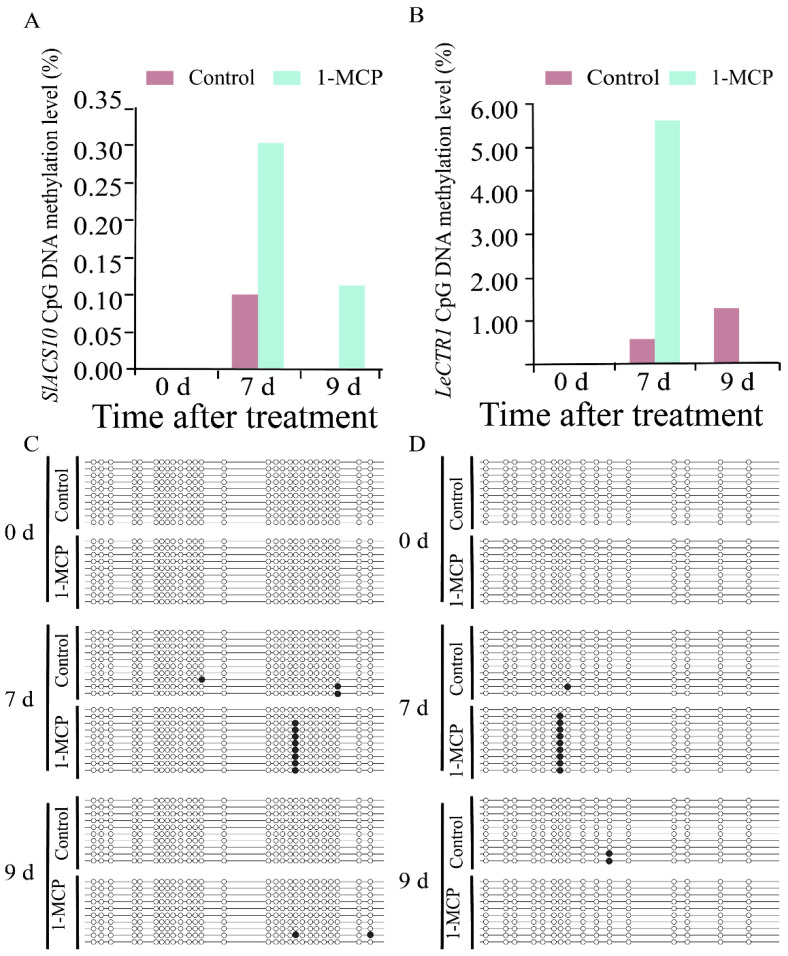
DNA methylation level and profile changes of *SlACS10*, *LeCTR1*, *LeEIN3,* and *SlERF-A1* genes in tomato fruit. (**A**) DNA methylation level of *SlACS10*. (**B**) DNA methylation level of *LeCTR1*. (**C**) DNA methylation profile changes of CpG islands of *SlACS10*. (**D**) DNA methylation profile changes of CpG islands of *LeCTR1*. Each line represents 1 clone, and 1 circle represents 1 CpG site. The black circle represents the methylated CG, and the white circle represents the unmethylated CG.

**Figure 4:**
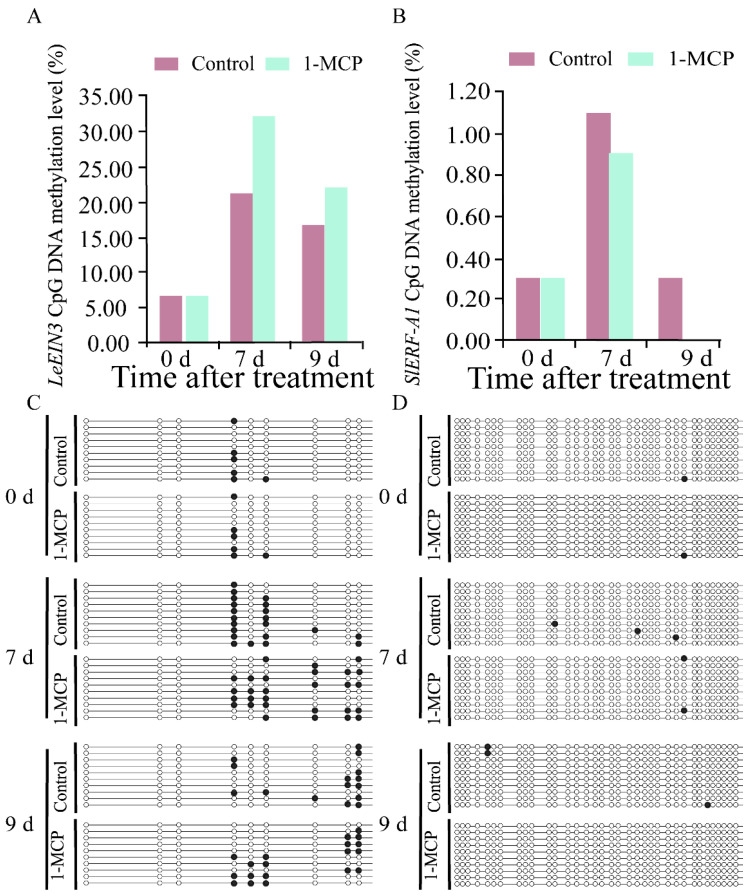
DNA methylation level and profile changes of *LeEIN3* and *SlERF-A1* genes in tomato fruit. (**A**) DNA methylation level of *LeEIN3*. (**B**) DNA methylation level of and *SlERF-A1*. (**C**) DNA methylation profile changes of CpG islands of *LeEIN3*. (**D**) DNA methylation profile changes of CpG islands of *SlERF-A1*. Each line represents 1 clone, and 1 circle represents 1 CpG site. The black circle represents the methylated CG, and the white circle represents the unmethylated CG.

The DNA methylation levels and sites of CpG islands of *SlACS10*, *LeCTR1*, *LeEIN3,* and *SlERF-A1* genes changed after being treated by 1-MCP (Figure 3 and Figure 4). As shown in Figure 3A, after storage for 7 and 9 d, the methylation level of CpG islands of *SlACS10* in 1-MCP-treated fruit was 0.30% and 0.11%, respectively. However, the DNA methylation changes of the CpG island of *SlACS10* in the control fruit were only detected at 7 d (Figure 3A). The DNA methylation level of the CpG island of *LeCTR1* in 1-MCP-treated fruit and control fruit was 5.60% and 0.60% after storage for 7 d, respectively; no DNA methylation changes were detected in the 1-MCP-treated fruit at 9 d (Figure 3B). Figure 3C,D shows the clones of *SlAC10* and *LeCTR1* with CpG island methylation sites located between the promoter and the first exon region (−45 to +309 bp) and the first exon region (+130 to +323 bp), respectively. DNA methylation testing was repeated every 10 organisms. The results showed that after 7 and 9 days of storage, the number of CpG island methylation sites for *SlACS10* in fruits treated with 1-MCP was eight and two, respectively. The control fruit had three CpG island methylation sites and two CpG island methylation sites on days 7 and 9, respectively (Figure 3C). The results showed that after 7 and 9 days of storage, the number of CpG island methylation sites for *LeCTR1* in fruits treated with 1-MCP was nine and zero, respectively. The control fruit had one CpG island methylation site and two CpG island methylation sites on days 7 and 9, respectively (Figure 3D).

As shown in Figure 4A, the DNA methylation level of *LeEIN3* in 1-MCP treated fruit was significantly higher than that in control fruit. After storage for 7 and 9 d, the methylation level of the CpG island of *LeEIN3* in 1-MCP-treated fruit and control fruit was 32.20% and 22.20%, and 21.10% and 16.70%, respectively (Figure 4A). The DNA methylation level of the CpG island of *SlERF-A1* in 1-MCP-treated fruit and control fruit was 0.90% and 1.10% after storage for 7 d, respectively (Figure 4B). After storage for 9 d, the DNA methylation rate of *SlERF-A1* in the control fruit was 0.30%, while no DNA methylation was found in the 1-MCP-treated fruit (Figure 4B). Figure 4C,D shows the clones of *LeEIN3* and *SlERF-A1* with CpG island methylation sites located between the promoter and the exon region (+92 to +286 bp), and the exon region of downstream (+334 to +677 bp), respectively. DNA methylation testing was repeated every 10 organisms. The results showed that after 7 and 9 days of storage, the number of CpG island methylation sites for *LeEIN3* in fruits treated with 1-MCP was 28 and 19, respectively. The control fruit had 21 CpG island methylation sites and 15 CpG island methylation sites on days 7 and 9, respectively (Figure 4C). The results showed that after 7 and 9 days of storage, the number of CpG island methylation sites for *SlERF-A1* in fruits treated with 1-MCP was two and zero, respectively. The control fruit had three CpG island methylation sites and three CpG island methylation sites on days 7 and 9, respectively (Figure 4D).

**Figure 5:**
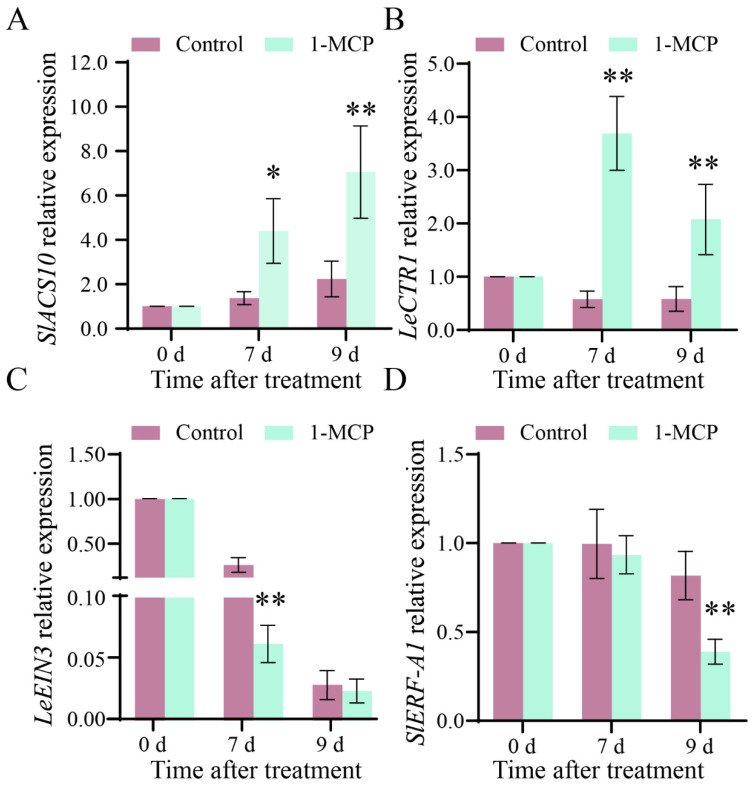
The expression level of *SlACS10* (**A**), *LeCTR1* (**B**), *LeEIN3* (**C**), and *SlERF-A1* (**D**) genes in tomato fruit. Vertical bars represent standard deviations of the means. Asterisks indicate statistical differences in the values at *p* < 0.05 (*) or *p* < 0.01 (**).

As shown in Figure 5A, the expression of *SlACS10* in 1-MCP-treated fruit and control fruit was significantly up-regulated during storage. During storage from 7 to 9 d, the expression of *SlACS10* increased from 4.40 to 7.05 in 1-MCP-treated fruit and increased from 1.38 to 2.24 in control fruit, respectively. During storage from 7 to 9 d, the expression level of *LeCTR1* in the 1-MCP treatment group first increased to a decreasing trend, while the expression level of *LeCTR1* in the control group remained unchanged. The expression level of *LeCTR1* in the 1-MCP treatment group was always higher than that in the control fruit (Figure 5B). During storage from 7 to 9 d, the expression level of the *LeEIN3* gene decreased from 0.26 to 0.03 in 1-MCP-treated fruit and decreased from 0.06 to 0.02 in control fruit (Figure 5C). The expression level of *SlERF-A1* in the control fruit was higher than that in 1-MCP-treated fruit during storage. After storage for 7 d, the expression level of *SlERF-A1* in 1-MCP-treated tomato fruit and control fruit was 0.93 and 0.99, respectively (Figure 5D). After storage for 9 d, the expression level of *SlERF-A1* in 1-MCP-treated tomato fruit and control fruit was 0.39 and 0.82, respectively (Figure 5D).

**Figure 6:**
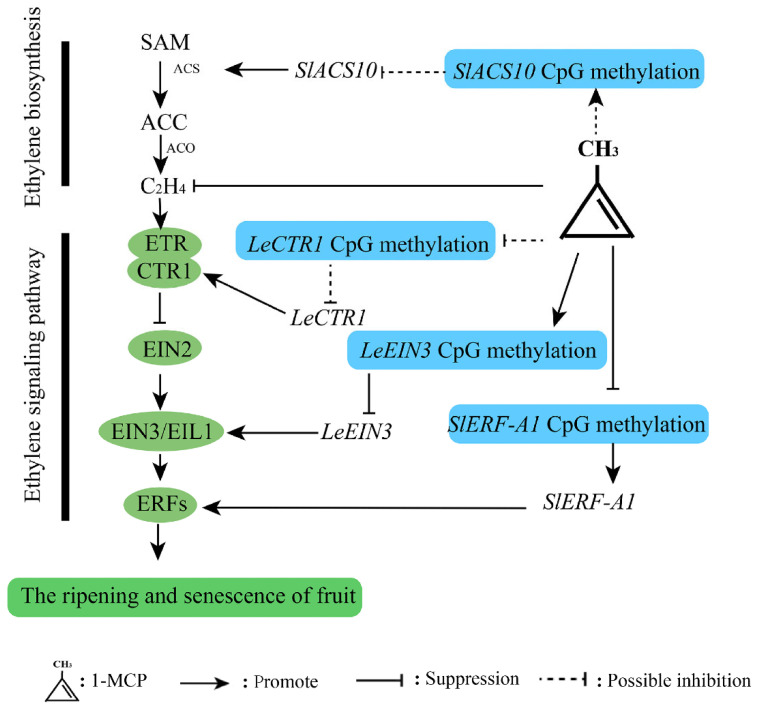
A simplified model for the mechanism of DNA methylation in the regulation of 1-MCP delaying postharvest ripening and senescence of tomato fruit.

In summary, 1-MCP can bind to ethylene receptors, thereby blocking ethylene signaling. In addition, 1-MCP may induce CpG island DNA methylation in the promoter region of *SlACS10*, which may promote the upregulation of *SlACS10* gene expression and enhance ACC synthesis. During the ethylene signaling process, 1-MCP may inhibit CpG island DNA methylation in the promoter regions of *LeCRT1* and *SlERF-A1*, resulting in unaffected gene expression of *SlCTR1* and *SlERF-A1,* hindering ethylene signaling. In addition, 1-MCP may induce an increase in CpG island DNA methylation levels in the promoter region of *LeEIN3*, leading to a decrease in gene expression of *LeEIN3* and a reduction in downstream *ERFs* activation, thereby blocking ethylene signaling transduction and delaying tomato fruit ripening and senescence postharvest (Figure 6).

## 3. Discussion

DNA methylation was considered to be a factor in regulating the ripening and senescence of fruit, mainly based on studies in tomatoes [29]. As a climacteric fruit, the tomato had a significant increase in respiration and ethylene production during ripening and senescence [30], which accelerated the accumulation of lycopene and the softening of fruit [31], causing a huge economic loss. 1-MCP is a cyclic alkene that can bind the ethylene receptor, block ethylene action, and delay the ripening and senescence of climacteric fruit. During the ripening process in citrus fruits, whole genome DNA methylation increases, with CHG and CHH methylation also involved, which may be related to the downregulation of DNA demethylase genes; the DNA methylation levels in hundreds of gene promoter regions are positively and negatively correlated with gene expression [26]. During the domestication and improvement process of pear fruit, the global DNA methylation level significantly increases, especially the CHG and CHH methylation levels, which gradually increase during fruit ripening; this suggests that this increase in DNA methylation level may be achieved by affecting the expression of related genes, thereby regulating the fruit ripening process [19]. Comparing the methylation levels of CHG and CHH during the development of “early-pedigree” and “late-cultivar” grapefruit, CHH methylation is the main reason for the differences in DNA methylation in fruits at different developmental stages. In addition, a high methylation of CHH was observed in the promoter regions of genes related to development and maturation [32]. Furthermore, DNA methylation typically occurs in regions rich in CpG dinucleotides, namely CpG islands. CpG islands are specific DNA regions rich in CpG dinucleotides, mainly located in gene promoters and exons, playing a crucial role in transcriptional regulation [33]. Their characteristic is high GC content (>50%), mainly present in the promoter region of genes [33]. However, there are relatively few reports on whether CpG island methylation changes affect postharvest ripening and senescence.

In plants, ACC synthase (ACS) was involved in ethylene biosynthesis, which converted SAM to ACC, and then ACC was oxidized to ethylene by ACC oxidase (ACO) [34]. Ethylene is perceived by ETRs, which initiates a signaling cascade by releasing CTR1 to block EIN2; this activates the *EIN3*/*EIL1* and *ERF* transcriptional cascade and, in turn, regulates ripening and senescence-related traits, such as color, the accumulation of lycopene, the softening of fruit [35,36]. So far, a total of 14 predicted *SlACS* members have been identified in tomatoes, among which *SlACS2* and *SlACS4* were found to be involved in the ripening and senescence of fruit [37]. In tomato fruit, 1-MCP treatment suppressed the expression of *SlACS2* and *SlACS4* and delayed the ripening and senescence of the fruit [38]. CpG Island Online Prediction (MethPrimer 2.0) shows CpG islands in the promoter regions of all 14 *SlACS* family genes in tomatoes, except for *SlACS11* and *SlACS10* (*SlACS12*) (Supplementary S2). Interestingly, previous studies have shown that the transcripts of the *SlACS10* (*SlACS12*) gene were almost undetectable during the ripening and senescence of wild-type tomato fruit, while the expression trend of *SlACS11* remained unchanged [37]. In the present results, compared with the control, 1-MCP treatment showed no significant effects on the DNA methylation level of the CpG island of *SlACS10* but promoted the expression of *SlACS10* in tomato fruit. The results indicated that the expression of *SlACS10* was induced by 1-MCP treatment, which might play a role in fruit ripening and senescence. In addition, both heat and melatonin treatment delayed tomato fruit ripening and senescence, and *SlACS10* expression levels were increased; the DNA methylation level of *SlACS10* CpG island had no significant effect [21,22], which was consistent with the results of this study. Therefore, we guessed that the DNA methylation changes of CHG or CHH sites in the CpG island of the *SlACS10* gene might also affect the expression of *SlACS10* and were involved in fruit ripening and senescence.

*CTR1* is a negative regulator in ethylene signaling. The *CTR1* loss-of-function mutations led to the constitutive activation of ethylene responses in both seedlings and adult plants [29,38]. In tomatoes, the expression of the *LeCTR1* gene was regulated by ethylene, and the expression of *LeCTR1* increased during fruit ripening [39]. *LeCTR1* silenced the tomato-induced expression of ethylene-responsive genes, such as *ERF5*, *EIN2*, and *EIN3* [40]. In the present results, compared with that in the control fruit, the DNA methylation level of the CpG island of *LeCTR1* significantly decreased in the 1-MCP-treated fruit, which increased the expression of *LeCTR1* and suppressed the ethylene signaling. In addition, both heat and melatonin treatment delayed tomato fruit ripening and senescence, and *LeCTR1* expression levels were increased, while the DNA methylation level of the *LeCTR1* CpG island was significantly decreased [21,22], which was consistent with the results of this study. This may be one of the mechanisms of 1-MCP delayed postharvest ripening and senescence of tomato fruit.

*EIN3/EIL*, as a positive regulator in ethylene signaling, was a nuclear-localized protein that was involved in mediating multiple ethylene responses [41]. EIN3 proteins bind directly to primary ethylene response element (PERE) motifs to regulate gene expression [35,42]. Several members of the *EIN3/EIL* proteins had been cloned and characterized [43,44]. In different species, the transcriptional level of the *EIN3/EIL* gene was differentially regulated by ripening, senescence, ethylene, and stresses [44,45]. In papaya, *CpEIN3a* was found to increase and participate in carotenoid accumulation during fruit ripening and senescence [46]. In the present results, compared with the control, 1-MCP treatment increased the DNA methylation level of the CpG island of the *LeEIN3* gene and reduced the expression of *LeEIN3* in tomato fruit. In addition, melatonin treatment delayed tomato fruit ripening and senescence, and *LeEIN3* expression levels were decreased; the DNA methylation level of *LeEIN3* CpG island was significantly increased [22], which was consistent with the results of this study. The increased DNA methylation of *LeEIN3* may have been involved in 1-MCP delaying postharvest senescence of tomato fruit.

Ethylene response factors (*ERFs*) are one of the largest transcription factor families in plants [47]. *ERFs* play important roles in metabolic regulation, biotic and abiotic stresses, and fruit ripening and senescence [48,49]. A total of 134 putative *SlERF* members were identified in tomato, which was divided into 12 subfamilies (A–L) based on their structural features [50]. A number of the *ERF* genes were inducible by ethylene and showed the expression pattern of ripening-associated [36,51]. *SlERF1* overexpression led to constitutive ethylene response and accelerated fruit ripening and softening [52]. *SlERF2* transcripts were the richest in tomato fruit and played an important role in ripening [53]. *SlERF6* played a significant role in fruit ripening by integrating ethylene and carotenoid synthesis pathways in tomatoes [48]. *SlERF.B3* was involved in controlling fruit ripening and senescence by regulating the production of ethylene and the accumulation of carotenoids [51]. In apples, the 1-MCP treatment effectively inhibited the genes involved in the ethylene signal pathway, including the *ERF* genes [54]. The transcript profiles of *SlERFs* genes in tomato fruit treated by 1-MCP and/or ultraviolet-C revealed that *SlERFs* played important roles in fruit ripening and senescence [55]. *SlERF-A1* underwent a steady increase in transcript accumulation throughout the wild-type tomato ripening [52]. The *SlERF-A1* (Solyc08g078180), *SlERF-C1* (Solyc05g051200), and *SlERF-D7* (Solyc03g118190) displayed increased expression at the onset of ripening and decreased expression at late ripening stages, which suggests that there is a potential role of these genes in regulating the ripening process [56,57]. Therefore, we predicted CpG islands for *SlERF-A1*, *SlERF-C1*, and *SlERF-D7* genes and found that only *SlERF-A1* contains CpG islands (Supplementary S2). Thus, *SlERF-A1* was selected as a candidate gene for detecting DNA methylation changes during tomato fruit ripening and senescence processes. In the present results, compared with the control, 1-MCP treatment showed no significant effects on the DNA methylation level of the CpG island of the *SlERF-A1* gene but decreased the expression level of *SlERF-A1* in tomato fruit. In addition, both heat and melatonin treatment delayed tomato fruit ripening and senescence, and the DNA methylation level of *SlERF-A1* CpG island was significantly decreased [21,22], which was consistent with the results of this study. The reason may be that 1-MCP treatment inhibited the *LeEIN3* gene expression, thus affecting the activation of the *SlERF* transcription cascade response and the expression level of *SlERF-A1*. Moreover, the DNA methylation changes of CHG or CHH sites in the CpG island of the *SlERF-A1* gene in 1-MCP-treated tomato fruit might also affect the expression of the *SlERF-A1* gene and be involved in fruit senescence.

## 4. Materials and Methods

### 4.1. Fruit and Treatment

Tomato (*Solanum lycopersicum cv.* Fen Gui Fei 455) fruit was collected at the mature green stage from a vegetable base at Demonstration Park of Modern Agricultural Science and Technology in Weifang, Shandong, China. The fruit was immediately transported to the laboratory and subsequently selected for uniform size, relatively consistent maturity, and no physical damage or disease. All fruit were immersed in a 0.2% (*w*/*v*) Sporgon solution (Bayer, Leverkusen, Germany) for 3 min to eliminate potential microbes and then air-dried at 25 °C. The 1-MCP (0.14%, SmartFresh™ Technology; Agrofresh Inc., Rohm and Haas, Spring House, PA, USA) was dissolved in a conical bottle containing sterile distilled water at a final concentration of 1 μL L^−1^. Tomato fruit was randomly divided into two groups and treated as follows: (a) the selected tomato fruit were placed in a polypropylene plastic box containing 1 μL L^−1^ of 1-MCP and then sealed with plastic bags; (b) the selected tomato fruit were placed in polypropylene plastic box and sealed with plastic bags. Finally, all fruit were stored at 25 °C and 80–90% relative humidity for 0, 3, 5, 7, and 9 d. The pericarp tissue was sampled, immediately frozen in liquid nitrogen, and stored at −80 °C. Each group was replicated three times, and the entire experiment was repeated twice.

### 4.2. Measurement of Pericarp Color, Ethylene Production, Lycopene, Vitamin C, and Total Soluble Solids

The pericarp color was determined and recorded (a*, b*, and L* values) according to the method of Li et al. [58], and the delta-E was calculated according to the method of Kim et al. [59]. The ethylene production rate was measured according to the method of Pu et al. [21]. Three replicates were used for each measurement.

The content of lycopene and vitamin C were determined using the Plant Lycopene ELISA Kit and Plant Vitamin C ELISA Kit (Jining Industrial Co., Ltd., Shanghai, China) following the manufacturer’s recommendations, respectively. Briefly, the frozen pericarp tissue was ground into powder under liquid nitrogen, 0.8 g of tissue powder was mixed with 1.64 mL of PBS (pH 7.4) in a 2.0 mL centrifuge tube, and the sample was homogenized for 1 min. The homogenized tomato was centrifuged at 3000× *g* for 20 min at 4 °C, and the supernatant was collected for lycopene and vitamin C analysis. The OD value was measured using a microplate reader at 450 nm (SpectraMax190; Molecular Devices, Sunnyvale, CA, USA), and lycopene and vitamin C content are expressed as mg Kg^−1^ fresh weight. The total soluble solid content was observed in a fruit juice with nine tomato fruits. Tomato fruit was homogenized in a grinder and filtrated with four layers of gauze; then, the total soluble solid was determined using a digital hand-held refractometer (PAL-1, ATAGO, Tokyo, Japan) and expressed as %. Three replicates were performed for each group.

### 4.3. Measurement of Cellulase and Polygalacturonase Activities

A powdered sample of pericarp (4 g) from 10 fruits was homogenized with a cold extraction buffer containing 3.28 mL of PBS (pH 7.4). The homogenized tomato was centrifuged at 3000× *g* for 20 min at 4 °C, and the supernatant was collected for cellulase and polygalacturonase activities analysis. According to the manufacturer’s instructions, the cellulase and polygalacturonase activities were determined using the Plant Cellulase ELISA Kit and Plant Polygalacturonase ELISA Kit (Shanghai Jingkang Biological Engineering Co., Ltd., Shanghai, China), respectively. Finally, the OD value was measured using a microplate reader at 450 nm. The activities of cellulase and polygalacturonase were calculated using standard curves and expressed as U Kg^−1^ fresh weight. Three replicates were performed for each group.

### 4.4. Measurement of Methylase and Demethylase Activities

The methylase and demethylase activities were determined using the Plant Methylase ELISA Kit and Plant Demethylase ELISA Kit (Gelatins Biotechnology Co., Ltd., Shanghai, China) following the manufacturer’s recommendations, respectively. The frozen tomato pericarp tissue was ground into powder under liquid nitrogen and randomly divided into two sub-samples to analyze the activities of methylase and demethylase. Briefly, the tissue powder (0.2 g) was homogenized with cold extraction buffer containing 1.88 mL of PBS (pH 7.4) and the homogenate was centrifuged at 5000× *g* for 20 min to collect the supernatant as the sample. The diluted standards and samples were added into 24-well plates, respectively, among which no samples were added to the blank control wells and incubated at 37 °C for 2 h with a plate closure membrane. The wells were washed five times with washing solution, the HRP-conjugate reagent was added, incubated at 37 °C for 30 min, and then the washing process was repeated. Finally, the chromogen solutions were added to the plate wells and incubated at 37 °C for 15 min. A sulfuric acid solution was added to stop the reaction, and the OD value at 450 nm was measured using a microplate reader. The methylase and demethylase activities were calculated based on a standard curve and expressed as U Kg^−1^ fresh weight. Three replicates were performed for each group.

### 4.5. RNA Extraction and Quantitative Real-Time PCR Analysis (qRT-PCR)

Total RNA was extracted using the Trizol reagent from the frozen pericarp tissues. According to the method of Wang et al. [60], the expression levels of *SlACS10 (Solyc08g079750)*, *LeCTR1 (Solyc10g083610)*, *LeEIN3 (Solyc01g096810),* and *SlERF-A1 (Solyc08g078180)* genes involved in ethylene biosynthesis and signal transduction pathway were studied by qRT-PCR. The sequences of the five selected genes are shown in Supplementary S1. The purified RNA was reversed transcription using TUREscript 1st Stand cDNA SYNTHESIS Kit (Aidlab Biotechnologies Co., Ltd., Beijing, China). The specific primers for qRT-PCR were designed using Primer Premier 5 (Table S1), and the qRT-PCR reaction system was as described in [61]. All reactions were performed in triplicate for each sample, and *GAPDH (Solyc05g014470)* was used as an internal reference gene. The relative expression levels of genes were calculated using the 2^−ΔΔCt^ method.

### 4.6. Bisulfite Sequencing PCR and DNA Methylation Analysis

DNA was extracted from the pericarp tissues using a Hi-DNAsecure plant kit (Beijing Tiangen Biochemical Technology Co., Ltd., Beijing, China). The *SlACS10*, *LeCTR1*, *LeEIN3,* and *SlERF-A1* genes containing CpG islands in ethylene synthesis and signal transduction pathways were selected by online software (http://www.bioinformatics.org/sms2/cpg_islands.html (accessed on 8 December 2024)) for methylation analysis, and their location in the gene sequence was shown in Supplementary S1. The purified genomic DNA was bisulfite-converted using the EpiTect Bisulfite Kit (Qiagen, 59104, Hilden, Germany) following the manufacturer’s recommendations. Briefly, 300 ng of DNA sample was mixed with 120 μL of the conversion reagent and treated as follows: 95 °C for 5 min, 60 °C for 25 min, 95 °C for 5 min, 60 °C for 85 min, 95 °C for 5 min, 60 °C for 175 min, and 20 °C for 50 min. Then, the DNA sample was purified according to the QIAGEN adhesive Recovery Kit manual. The modified DNA was PCR amplified for 40 cycles with gene-specific primers (Table S2). The PCR products were cloned into pGEM-T vector using the EZ-T Zero pTOPO Cloning Kit (GEnStar, T180-100, Beijing, China) and sequenced. The DNA methylation status of the sequences was analyzed using a BiQ analyzer. All reactions were performed in triplicate for each sample.

### 4.7. Statistical Analysis

All data were analyzed using a one-way analysis of variance (ANOVA). Mean separations were performed using Duncan’s multiple-range test.

## 5. Conclusions

In summary, the expression and changes of DNA methylation of the CpG island of *SlACS10, LeCTR1, LeEIN3,* and *SlERF-A1* genes in tomato fruit induced by 1-MCP might play important roles in postharvest ripening and senescence. The present results provide a foundation for genetically modifying the epigenetic target sites of ethylene signaling genes and delaying postharvest ripening and senescence of tomato fruit. In addition, the results of this study provide the possibility for future genetic engineering and epigenetic modification to develop new strategies for manipulating tomato fruit ripening and senescence. Moreover, this could lead to the creation of tomato fruit with an improved shelf life, enhanced resistance to postharvest diseases, and better overall quality, which would be beneficial for both farmers and consumers. Furthermore, the understanding of the mechanisms underlying the effects of 1-MCP on DNA methylation could also be extended to other crops, contributing to broader advancements in agricultural science and technology.

## Data Availability

Data are contained within the article or Supplementary Materials.

## Supplementary Materials

The following supporting information can be downloaded at https://www.mdpi.com/article/10.3390/ijms26010168/s1.
