# Supplementary material for: DNA Methylation Is Crucial for 1-Methylcyclopropene Delaying Postharvest Ripening and Senescence of Tomato Fruit
Source: Int J Mol Sci. 2024 Dec 28;26(1):168. doi: 10.3390/ijms26010168 (PMC11720368; doi:10.3390/ijms26010168)
Supplement: Supplementary file 1 [file ijms-26-00168-s001.zip › Supplementary S1.pdf]

SUPPLEMENTARY S1

ATG in bold indicates the start codon.

Red indicates CpG island.

>SlACS10

-208 AACCAAAAAA AAAATAAAAA AATTTAGGCG GACAAAAAGA TAATAATAAT TCGACCAACT
-148 ATTTGCCACC ATTTTCTCCA AATCTCTTCT CCATCACTCA CCTCCCTTTG TATTCTCCGT
-88 CCACCACCAT CTCGCCGGCC AGGTAGCTGA CTGACGTCAT ACTCCGACAA GGTGACGTCT
-28 TTCTCTACGCCTCCACTATG TCACAGTT**ATG**ACAAGGTCA CGGAACCGTT CACCAACCAG
33 AACAACAACC ATTTCAACTG GCGGCGCCGG CGGCAGAGAC GGAGGAGGAG CCACGACGGC
93 GATGAGGGTG ATAGTACCCT TACAAGGTGT AGTACAAGGT CGGGGTGGTC TTTTCTTAGG
153 TTCAGTAATA CCCTGTGCTC TTTTTTATTT TTGGCAACTC TACCTAAAAC GAAATCGTTC
213 CTCCGGTGGT GACAATAACG GCGAATCTAC GGCACCGGCG AGGTCACCGT CGTCGACCCA
273 TTTGCCAGAA GTGTCTTCTG GGTCTGGGTT ACAAAGGGTT CATTCACGTC TGTTATTGTC
333 ACCGAAGGGA ACTACTGGGC AATCTCAGGT ATCTGCTAGA GCTAATTCGA TTATTTCTAA
393 ACAAATTGAT AGTAGCCCTT ACTATGTTGG ATTGAAAAGA GCTTCTGAAG ACCCTTATGA
453 TGAGTCGAGT AATCCAGATG GTGTTATTCA GCTAGGATTA GCAGAAAACA AGGTAATAAT
513 TTTGAATATT TGGATATATT GAATCATTTT CTAGAGAAAT GTACCAAATG CAATACGTTT
573 CTTGTTGGGA AGTGAATTTT TTTCCTTTCT ACTTTGGTTT GTTGTTTTGA TGATTTTGAA
633 TTTTGTCTCT ATTTGTATGG TTACAGTTGT CACTGGATTT AGTTCAAGAA TGGCTAGCAG
693 AAAATGTGTC AAGATGGATG ATGACTCAAG ATTCGAGTAT AACCGGAATA GCTACTTATC
753 AGCCATTTGA CGGGTTATTG GAGCTGAAAG TGGTAAGCTT TTTAGTTTTT CCTTGCATAC
813 TAAGATGGTT CTTCTTGAAC TATTCAATTA CTAGTAGTTG TTCATATGGT TAATAGTTTT
873 TACAGGCTAG AGAAGGAAAT TGAACAAAAA AATTGCTTGA AAAGGTTTGT AAAAACTTAA
933 AGTTGGTAAT TTGAATCAAG ACTTTATAGG AATAAATCAA TGAGATACCT TTTCAGGTGG
993 TATTAGTTTA TAATTATTAC CTAGGATGGA CTCATGTCAT GTCTATATGC TTGTATAACA
1053 GTTACTCCAT ATCAATTATC ACCCATCCTC TAGTTCTGTG TGCTTTATCG CTGATTTAAT
1113 TTGAAATGTG AATCCCTGGC TGTGGATCGG TAATGGCATT TGACAATCCC AAAGTTACCT
1173 TTCGTATACA AGGGTATTGT ATGTCCCATC TTGAGCTTTC TAAGTGTATA AAGTACAACA
1233 GACATAAGGA AAAGCAATGG TAAGCTTCAC TTTCATTTTA CAGTGCAACT GACAATTAAC
1293 TTCTGAAAAG TTTGTCAACG CTTTAACTTC TACGCTATCG ACGCTAATTA TCATTTTGGA
1353 AAAATAAACA CTGCTCTGAA TGTTTTATAG AAATGATAGG AAAATTTTGA AACCTGAGAA
1413 TGTGTGTGAC CAGACAAAGC ACCAAAACCG TATCCAGAGG GTAATTAAAT TGTTAAACTT
1473 CTCCTACTTG AACATTACAT TGGTAAAACT TGAGTGGAAA CACGTAATTG TTGTGGATCT
1533 AGCTCAGATA TTTATGTTGT TTCAGCTTTT GATAATTGTC TATGGTGGTG TTTGTGCTGA
1593 CCAGAAAAAT AGGACCTTGC AGTGTGATAT GATAAAAAAA TCTAGATTTC GATATTTCAT
1653 CACATTAGAG AATAGTTTGA CCTGCTTTAT TCTGCATGGT TTTTGTTATA AGCAAGTTAA
1713 TAGTGAATAA TGGACTCAGT TGTTCTGAGA GGTGGCCTAT TATTTCCTTG ATTAATACTC
1773 ACCTTGATAC TCATCACTGT GTAACTTATC GAAGGACTAT TGCTAGATTT GCCTGTTGAA
1833 GATGGAAGGC AATTTTGGCC TCTTTGAAAT ATCAGTTGGC TACCTTTGTC ATCAGGTATC
1893 ATATGGGTGG GAGATGATCT GATGGATTTG CCTTTTAAAA TTGCGTGTTT GAGAATTGTA
1953 TGACAGCAAC ATATGAAATT ATCTTTGAGC CACTATGATG ATTGATTCAT AAAAACAATA
2013 AGTGTTTTGT GTGATTCTAC ATAATTATGG AATCTCTTTG AAATGATTTT TATGTTTTCT
2073 CACCTGTATG CAGGCTGTGG GAGAATTTAT GTCTCAAGCT CTAGAGAGAT CAGTGTCCTT

2133 CAGCCCCTCA CAAATGGTTC TTACCGGTGG TGCAACTCCT GCGCTTGAGA TACTGAGCTT

2193 CTGCCTAGCT GATCCTGGAA ATGCTTTTCT TGTTCCCTCA CCATATTATC CTGAGTAAGT

2253 CTCATTCTCC CTCAAAGAAA TTTCGATCTT TTGGTCATTT TTACGTATTT AGTGTATAAA

2313 ATGAGCTCAA TGCATTTGCA GTCTTGATAG GGATGTCAAG TGGAGAACTG GAGTGGAGAT

2373 TATACCTGTT CCTTGCCGCA GTGCAGACAA CTTTAACTTG AGCATTGATG CTCTTGACCG

2433 AGCTTTTAAC CAAGCTAAGA AACGTGGTCT TAAAGTACGA GGGATTATCA TTTCTAACCC

2493 CTCGAATCCT GTGGGCAATA TTTTCTCTAG GGAGACACTT TATAACCTCT TAGACTTTAC

2553 AACTGAGAAG AACATCCATG TAATATCCAA TGAAATCTTG GCAGGGTCAA CTTATGGAAA

2613 TGAAGAATTT GTCAGCATGG CAGAGATAAT TGATTCCGAA GATTTTGACA GGAGCAGGGT

2673 ACACATTGTG TATGGTCTCT CTAAGGACCT TTCTCTTCCT GGGTTCAGGG TGGGGGTCAT

2733 CTATTCTTGC AATGAAAATG TTCTAGCTGC TGCGAAAAAA TTGACAAGAT TCTCATCCAT

2793 TTCAGCCCCA ACACAGCATT TAATCATCCA AATGCTATCA GATGCAAAGT TTGTACAACA

2853 ATTTATCAAA AAGAACAGAG AGAGGCTAAG AAGGATGTCT TCTCTATTTG TCAGTGGATT

2913 GAAGCAGCTG GGAATTGAGT GCACCAGAAG TAGTGGGGGC TTCTATTGTT GGGCTGACAT

2973 GAGCAGGTTA ATTCGGTCGT ATAATGAGAA GGGTGAGATT GAGCTTTGGG ATAATCTACT

3033 AAATGTAGCT AAGATCAACG CAACTCCTGG TTCTTCCTGC CACTGTGTTG AACCTGGGTG

3093 GTTCAGGTTA TGTTTTTCGA CATTAAGTGA GAAGGACATT TCCGCAGTTA TGCAACGTAT

3153 CCAGAAAGTT TTGGAGTTAC GTAAGTCTCT GAGTTAAGTT GCTAATGATA TGATCCCTTA

3213 CATTAGTCAA CCGTGTAATC ATAGGTCCAA AACTTCTGCA GAGATTTTAA GGGTCGAGTG

3273 TTGTGTGATT TTAATGGATC CATAAGTTAT CAGTATCCTG ATAATGAGGT GAGGATGCAG

3333 CAGCTCTGAG AACTAAATGC AGAGAAAACA AAATAAAAAG GCCAAATTGT ATGCAGATGA

3393 TTCCAGAAGT GTGTTCTTGG TCCTCTCCAA CTTTGTCATT TTCACAAATG CATTCCTCCT

3453 CCCAGTTTAT GGATTTTGAA GATAGGTAAA TTAGCAGTCA AAGTTTATGC AAGGAAAAAC

3513 ATATAGCATA TAGAAATGGC TTCAAATGAA ATCCATTTCC AAATTCTTTG TGCATAGTCA

3573 ATAAGTAAGG TCTTAAATCT TGATATTTTC TTATTATTTC TCATTGTTGT ATAGTTACAG

3633 GTAGATGTAT GTACTCATTA TTTGTTCACA TTCATTCTTG TGTTGGCACT AGATATGTAA

3693 TTCGATAAAT GAATCAATTT CTTTCATCCC TT

＞*LeCTR1*

-261 CTTCACGTAT TTACGCACAA AATTCTATGC ATCTTTCAGC CTCATGACCA AACAGCCCCC

-201 ACTTGCTATA ATTAATTCAA GTTAAAGTCC ACAATTCTAC TTTAATAGCT TTTTTACACC

-141 TCAAAATTTC TCAGAATATT ACAAACACAT CCATTTTCTC CATCCTCTTT CTTACTGTGA

-81 GAAAATTTAG AGAGAGAGAA GTGTATTTTG AGCTGAAAAT TGCTAATGGC AGTTGGTAAC

-21 TGAACGTTTT TATACAGGGA A**ATG**TCTGGT AGACGATCGA GTTATACTTT GTTGAATCAA

40 ATTCCTAATG ATAATTTTTT CCAGCCGCCG GCGCCGAAGT TCTCTGCCGG AGCTGGTGTA

60 GCGCCGTACG GTGAGTCTAG TTCTGCTGAG AAGAACAGAG GTAAAGTATT TGATTTGGAC

120 TTGATGGATC AACGCATGAT GCAATCACAT AACCGGGTCG GATCATTTCG GGTACCGGGT

180 TCGATCGGGT CGCAGAGGCA ATCAAGCGAG GGTAGCTTCG GTGGTAGCTC GTTATCTGGG

240 GAGAACTACG TGGGGACTTC CTTTGGGCAT AAGAATGAGG GCTGTGGCTC GTCGGTGGCA

300 AGGAGCTGGG CGCAGCAGAC GGAAGAGAGT TATCAGCTGC AGTTAGCTTT GGCAATAAGG

360 CTCTCTTCAG AAGCAACCTG TGCTGATAGT CCAAACTTCT TGGATCCTGT GACTGATGTA

420 TTGGCATCCC GAGATTCAGA TTCTACTGCA TCAGCGGTAA CAATGTCACA TCGATTGTGG

480 GTATGACATT TATTGCTAGG AATCCTTTGA TAATTTCATA TTGGTAAAAG AGAGAATTAA

540 GAGTAATTGT TTAGTAATGA TTTTCAGCTT TACCAACAGT GAAGGATGTA GTGTAGAAGC

600 TAGTTGGCTC TAACTGGTGT ATACTTTAAA GATTCATTAA ATAAATACAA ATAGTAGACT

660 TTGAACTTAC TCAATCCATA TAGTTTTGAA CTCAAACTCG TAAAGTTCAA TCGTGGATCC
720 ACATATGATT ACAAATGTCT TAGCTGATTA AGTGGTGGAT TGTGGGAGGC TTTGAGTTTC
780 TAATCCAAAA ACTTAAGGTA ATAGGTGAAA TGGTCAACAC CCTTATAATC ACGTAGAACA
840 AGAAATGACT ATATATCATC TCACACATGA ATTACAATAT GGCCCATAAG GGTGTTAGGA
900 TGGGCATTTC AAATGTTGGC TCTGATTTGT CAAAAAATTT GATTATCCAA CCTAAATTCT
960 TTTGACATTA AGTGGAATTA TCCGGGACCT TTTTAAACTC TTTAAGAAGT TCTCAGACAA
1020 CCTATGCAGG ACAACAATAT AACTCAGTTT AGGATTTAGT TTAGACTATT AACGTCTCCA
1080 TGCATAAAGT AGGTGATTAA GCGGGGAAGG AATGTGCTTT TCAAATGCAA ATACATGCAT
1140 AATTATTATA TATTGTAATT GGTGGATCCT TTGTTAGAGT CCTACGTTGA TCAAATATTT
1200 TATATTGAAG TCTCTCTGTA TGGTTTTGGA CAATTTTCAC ATTATAAACT AGCTTTCGGG
1260 GTGAGTTAGA CCCAAAGTAT ATTCATCATG TTACAACTGG ATCCATCCCT TTTTATCATG
1320 AAAAAAGTTC ATTAATTATT CTTGTAGTGT CTTGATTTTG ATTCCTAGCA TCATCTATTG
1380 TTTATTGTGT TTCTGCTATC GCACTATTTT GTTGTTGTTG CTGTTTCCTT ATTAGTTGTT
1440 ATGGTTCTTC ACTGCTTTTC CTTTTCCTAC TTCAATTTGT TGTACTCTAG TTGAGAGTCC
1500 TCCTGAACTC TGCCCCCATG AGATATGAGT ATGATCTGCA TACACTCTAC CCTCCCAAAG
1560 CTCACTTGTG GGATTTCAAT GGGTATGTTG TTGTTGTAAT AATTTTTTAT TAATAATGGA
1620 CAAAAAATAT CAATATACAA GAAGTATACC AAATTGTACA AAAATCTAAA ATATTTGTGA
1680 ACCTCACCTT ATTTTCTAAT GCTTAAGGCT CTCGGCTGAG TAGCAGGAGC AATTTTTAAT
1740 TTATTACTTT ATGACGAGCT CAAACGATGG ATTCAAAATA ATTTCTTGGC AATAACTTTG
1800 TCCACTGAAT AGAAATATTA ATCACATGTT CCATAGTCCT GGGGCTATCA ATTAGCCAAA
1860 CACATGGAAT CCTGCTCTTT CATAAATTCT CAGTTGTAGA GTAACCCAAT GTTGGACCTG
1920 TATAGTAACC CAAAGTTGGA CCTGTAGTCC ACGCTCCAGA GGTCCAACTC TGCTATGTGG
1980 TTGTGTGTTA ATTCCATATT GTTTGAAAGT GGGGATTGAG TTGTGCGTGT CTTAGAGTTC
2040 CAGATGGTTG AGAATATAGA CTGTATTCTC TTTATATGGT TTTATATAAT TCTTAATGCT
2100 TAAACTAGCT CTTGGGATGG AGTTAGGCCT ATTGTTCATT TCTTTAACTT CCTTCCCAAT
2160 TTCAAGAGAT CTTAATAATT CTACTCACAA CGTCTTTATG TTTATACCAT GTGTCAAGAC
2220 TCTTAAATCT CTAAAATTTG ATATGGAGGT GAAAATAATT GATTGCTTGT GACAAGGGAA
2280 GGATGGCCTA GTTGTAATCC ACACCTTCAA CTCTAAGGTT GGGGGTTCAA GTCACTAAAG
2340 GAGCGAAGTG GGAAAGTACA ACTGATGGGC TCTTGCCTCT TGGGTAGGGC TGTAGGAAGT
2400 CAAACTTTTC TAAGTGCTAA TCGCGATCAC CCCTTTTCTT CTTTCTACTT TTCTATTTCT
2460 TCTTTCATTG TTGCCAAATA CAATGAGGCT GAGCTGTTTA CACTAGCTCA AAAGTTTTAA
2520 GCCTTTCTCG TAGTCAAAAT GTGATCTTGG CATTGACAAC CCCCTTCCTT TTTTCTGCAA
2580 GGCCCTAGAA AATGATATGA TAAAAAAAAA GTTTGATCAC ATATTCTTCT GTTAGTTTCA
2640 TGTTTTGCGG TAGGAGCTAC TTCATACTAC CTTGATGTTT TTTTGAATGT AGTTATACTC
2700 ACCTATTGAC TAGATGTTGC CTTTCTATTG TGATTTTAAA TTATCTTACA GATCTCTTCT
2760 CATTCTCAAT TGAGAGTTGA GACAGTCTCT AGATTTCCAG GTCATCATTC CTAATGTCAT
2820 TTTATTGCTC TGGCCTCTTT ATTTATGCCA GATAAATGGA TGTATGTCAT ACTTTGACAA
2880 AGTCCCTGAT GGATTTTATT GGATTTATGG GATGGATCCA TATGTTTGGG CTCTCTGCTC
2940 AGTTGTGCAA GAAAGTGGCC GTATTCCATC AATTGAATCA TTGAGGGCGG TTGATCCCTC
3000 TAAAGCACCA TCTGTTGAAG TGATTTTAAT TGATCGCTGT AATGATCTCA GCTTGAAGGA
3060 ACTGCAGAAT AGAATTCATA GCATATCCCC TAGTTGCATC ACCACAAAAG AAGCTGTTGA
3120 TCAGCTTGCC AAGCTGGTTT GCGATCATAT GGGGTGAGTA CTGAAAGCTT TTTCTTAGAA
3180 GCTCCAACAG ACCCACTTTT TTCCTATTTC TTTTTGTGAT TAGCTTTATT CTCTCTATAA
3240 AGGGGTGCAG CTCCTGCTGG AGAAGAGGAA CTGGTTTCCA TGTCAAAGGG GTGCAGTAAT

3300 GACCTGAAGG ATCGTTTTGG AACTATCGTG CTTCCCATTG GTAGCCTGTC TGTTGGGCTT
3360 TGCAGGCATC GTGCTTTGCT TTTTAAAGTA TGTACTTGTT AAGTTGGTTC TGGTATAAGT
3420 CAGTATATCT AATTTGTAAA GGAAAGATTT AAGGATAAAA TCAGACTAAT GTGTGTATTT
3480 ATCACTAAAT AGAAACCTTG TGGATATTGT ATTTATTTTT TATTTATTCT ACGCTTAGAG
3540 TGATATTGTT TGCAATCCAT AGTGTCTTAG CTTGGTTGTC ATGTACAAGG TTGATGTTTT
3600 ATCATTAACA TCTATGTAAT CATTACAGGT GCTAGCTGAC ATCATTGATT TACCATGTCG
3660 AATTGCCAAG GGATGTAAAT ATTGTAATAG CTCTGATGCT TCCTCATGTC TAGTTCGATT
3720 TGAACATGAC AGGTAAAAGT TCAGATTACA TATGGTGTGT ATTATAATCT TTGGTTCATT
3780 TGAAAGAAAT TTTACTAGGA TATAAAGTGG GAAAGATACT CAATTCACCT TTTGTATACC
3840 TTTTTCCTAG ATAGAATAGT TTTGGGGATG GTGTGTTGAG TCCTTAAACG AATGGTAGTT
3900 GATGCTGCCA CTTACTTTTA TTTGGATAAC TGATACCCCT CATATATATT GTATGAAATG
3960 ATCAAGATTT ACTTTCCTAC TGCTATAATG TGTCTGCTGG TAACATTGTA ATTTGTATAG
4020 ATGCTTTCTG CCTTCTTTCA CGTGGTTATT TTATTCATGC TATGCCCAAC ATCCAATTTT
4080 TCTTCGGTTT CATAAAGCTG GTGAAGAAAA ACAAATTCAA AAAATGATTT ATTTGTCAAA
4140 AGAGCTGCTC AATATATATT CGGAACATGA CTGGCAAAAT TTCTCGTTGT AATAGCTAAT
4200 AATGTGACCT TCCTGATGTG ATAATATATA TCTTTAAAGA TTCAGTCGCT AGCAGAAGTT
4260 TGCTTCTCTT AGTACTTGCT GCAGCATTCT TAGTAATTTA GAATTAAGAT ATTTGAGTGG
4320 AATGGTAGTT CTTACTAATA TGCAGAGATT GAATTTTTTA AATGAAACTT GAAAAAACGG
4380 ATCCACTGTT GATGAGATAG GGAATGTACC TAAAGTTGTC ATACGGTCTT AATCACTCAG
4440 ATTTTAAATC ATGATCCAAA TTCCAATGCA AAAAAATCCT TCTCTTCTTC CATGCAGCCC
4500 CAAATGCAGA TAAGAAGAAC CAAAGTACTA GAAGGAAGTA GAATCAGATA TCCAAAGCAG
4560 ACTGCTACTT TAAGTTGGAA ATAGATTAGT AACTGGATAT GTCTGTTATT TTCATCTGTT
4640 GAGCTTTTCT GCTGGATTCA GTTTACATGA TAAGACATAT ATAGTTTTAA AATATGCTAA
4700 ACATTTGTGT GCTACCTGGT TAGCCTCATA TTAGGAAGGA GTGCTAGTAT TGACATGTAA
4760 CTCTTGACCA TACAGGGAGT ACTTGGTTGA TTTGATCGGT AAGCCTGGAG TTTTAAGCGA
4820 ACCAGATTCC TTGTTGAATG GTCCATCTTC CATCTCAATC CCTTCACCTT TGCGCTTTCC
4880 GAGATACAGA CAAGTTGAGC CTACAACTGA TTTCAGGTCA TTGGCCAAAC AGTATTTCTT
4940 GGATAGTCAA TCACTTAATC TACTGTTTGA TGATTCTTCA GCTGGTGATT ATCTAACTCT
5000 TTAGCTGCTT AAAAAATGCA TTGTATTTTG TTTGTTTTGA ATTGTCCTTT CCCAACATCT
5060 TACTTGGCCC TTGCCATGAT ATGTTGACAA ATATGCAGGT TCATACACAA ATTTAGAATT
5120 GCTTCAGTCT AGCATGCTGC ATTCCACCAA AATGCCCCCT TTTCCCAAGT TCCCTTTCAT
5180 ATTGTGTCAA AATTGTGGTG TCTGATAATC AGTTGAACTT ATTAGAATCT CCTGAAAAGT
5240 TCGCCAGTAT GTGTGGTTAT TTGTCTTACT TGAACGGAGT ATACTTTCAG CTTGCTTATA
5300 ACATTGTTCA ACACTGTAAT GTCTTAAAAC ACAATTGAGT ACATTGACGA TCCAGAGTTT
5360 CAAGTTTTTT TTTGGAACAG GTGAAGTGGT GTATATTATA GAATAAAATT AGAAACCTGG
5420 TGTTAAGCTG GTTCCTTGAT AAGTACAAAG TGTGAAACCC TTCTGTACAA AAACAAAAAG
5480 AAGGTTCTCT CACCAGACTA AGCATCTATT CCTGTAGTGA ATCTATGAAC TCTAACGTAC
5540 CATGTTCATC TTTAACATAT TGTAAGGTAC ACCTCGAGCT AGTCCAGATA CCCCATAATC
5600 GTGCATCCAC ATGAGAATGC AATAACAGGT GGTCCACATT TTCTCTTGAA CATCTGCACA
5660 TAAAGCACTA GCTAACATAT GTGATCCTCT TTTTCCTCAA GTTTCCAGTG GTCAGAACTG
5720 CTCCCTTGCT GCCAACCAAG CCAAGCACAC TTTTTAGGTG TGCTAAGAAG CCAAATAATA
5780 CATGCAGGAA GCCCATCTTT CCTCACTAAA AGCTTGTGGT AGTATGATTT TAATTGGAAA
5840 ACCTCCATCT ATCCTTGCTT CCCACCTCCA CAAGTCCTAT CTACTGAGTG TTGAGTTTTG
5900 TTTCTAGAAA ATTTTGATCA ACCTTTGGAA ATTCATCAGT ATATATACAT GTATATATAC

5960 ATACTTCGCA TCTTCCTTCT TTTTTTCTGG GACAACCTAA ATTTATAATA TGCAAAGGCA
6020 AATTAATAAC ACTAGTGATC TACCTTGTGC CTTGACTTTT ATCTAGGAAA GTATGTAAAT
6080 AAAGTATGAA ATCTATTTTT TTTTAATTGT GCAAATAAAT ATTAGTACTA TTTTGTTTTG
6140 GACGATTTCT CTTCAGTTTT TTTAGTGAAT ACTAGATCCA TCCAGTCCTG TTTCAGAGAT
6200 TCATAATGAT TAGTATTTGA TGTAGGAGCT GCAGCTGATG GAGATGCAGG ACAATCAGAC
6260 AGAAGTTGCA TTGATAGAAA CAATGTAGTC TCTAGTTCAA GTAATCGTGA TGAAATTTCT
6320 CAGTTACCTC TGCCTCCATT AAATGCATGG AAAAAGGGAC GAGATAAAGA ATCTCAACTT
6380 TCTAAAATGT ATAATCCTCG AAGTATGTTA AACCCAGTGA ACATGGACGA GGACCAGGTT
6440 CTTGTGAAGC ATGTTCCTCC ATTCCGGGAA GATGCTCAAT CACCGATGAC ACGACCAGAT
6500 ACAGTAAATG ATACTAGGTT TCTTGCTGGA GGAGGTCATG TTGTTTCTGC TATACCAAGT
6560 GAAGAACTTG ATCTCGATGT AGAAGAGTTC AATATTCCAT GGAATGATCT GGTTCTAATG
6620 GAGAAAATTG GGGCAGGTAA TCTGTCTCCT GGATTTTAGC CATTTGAATA GTGAGCTGTG
6680 GTTGCTTGGT TAAATGTCAG TTATTTTCTG TTATTTTTTA CCCCTTGAGG GGACACATTA
6740 TATGGGATTG TATAGTACCA TAACATTCTT ACTTGATTAA GCTAACATAG TTTGTCTATT
6800 TTCAGGGTCT TTTGGTACTG TTCACCGTGG TGATTGGCAT GGCTCTGTAA GATACTTATC
6860 CAATTAGATC TCAGTTTTTG GAAATCTTCT ATCTGAAAAA ATATCTCAAC GTATCTTCTA
6920 CTTAAGAGAT CCATTTTTGC AATGTGAATT TTTTCATCTC TATTGTACTT CTTTGGGGGG
6980 AAACCGTATT TTTGTTGTAG TTTAATCGAG TTAGCTCTGA AGCTCCCTCT CAAATACTTT
7040 TTACTGGCGT TGTTGTTTCT TTTTCTCTTT CAGATTTTCA CTTTTTTATG TTGGGGAGGA
7100 GGGGTAGTCT ATTTTATGTC TAGTGGAGAT ACAACCATGT GAAGCCTGCT ATATTACGTT
7160 GGTCTTATTC CTTCCTGATT GTATCTCATG GAAGTTTGTA TCATTTAGGA TGTTGCCGTG
7220 AAGATCCTCA TGGAACAAGA TTTTCATGCA GAGCGACTCA AGGAATTTTT GAGGGAGGTA
7280 AGTCTGTGCT TTCTCCCCCA ACATCCGACT AGCAATCAGG AAGAACTAGA AAGGGACCAG
7340 AATGCACGGA GATTGAGGAT CTGCGGACGT CAGTTTGTTA TTTGTATTCC TTCTACATCC
7400 TTCTAATTCT GTTATTTTTT TGGGTGTCCT AGGTTGCAAT TATGAAGCGG TTGCGACATC
7460 CAAATATTGT ACTTTTTATG GGTGCTGTCA TTCAGCCACC AAATTTGTCC ATAGTCACGG
7520 AATATTTATC GAGGTCTTAG CCTATTTCTC TCTTCAGCTT ATTAGAGAAA GCACTTTGTT
7580 TTTCCTGATT TAGCTGACTG GTTGCCATGT TATCATGTAT TTCAGAGGTA GCTTATATAG
7640 ACTTCTTCAT AAACCTGGTG CGAGAGAGGT GTTGGATGAA AGGCGTCGCC TGTGTATGGC
7700 TTACGATGTG GTATGATAAA TGTGTCCTAC TCCTCTCTTC TTTCTCATTT TACTTATTCA
7760 CTGGGGCTGA TCGCTTAATA TGATACCATG CAGGCAAATG GGATGAATTA TCTTCACAAA
7820 CGCAATCCTC CCATTGTGCA CCGAGATTTA AAATCTCCAA ATCTTCTAGT AGACAAAAAA
7880 TATACAGTGA AGGTGAGAGT AAGAACTTTA GCAAGCATAA AGGTTCAATT TTCTTTGTCA
7940 TGATTGATGT ATTCTCAGAC TTGGCTGTCA GAGTAGTCAA ATCTGAAGGA AGCATCATCC
8000 TGGATAGGAC ATTTTTTTTT CCATTTGAGA GGCGTCTTCA AACCCAACTT CCTCAGACAT
8060 TTTCCCAAGG GTCAAGAGAA GCATATAAAA GTTGTAGTTT CAGAATCTTT ATAACCTAAT
8120 CAAAATTGCT GAATAACATA AAGCAAGTAT TTATCAGTTT CTCTGATGAA AGTTAACTTT
8180 TCATTCCATG AAATGAGTTA TTTTTCGATA AGAGAGGATA TTTCTTGAAA ATTCTGTGAA
8240 AAGGGGAAAA ATAATTATAC ATTATTTGAC ATTTTGGAGG AAGTCTGGTC CATAATCTTT
8300 CTTTGTGATC TTCTAGATAT ATGCCATCTA TAAAGTGCTT ATTTCAACTG CCATTTATCC
8360 AGCATGTTTC AAGAAATTTA GGAGTCAATA TGAGAAGGAA GTTTCTATTT CCGGTTTAGA
8420 TTGTCTTTCA AATTTTCTTC TTATGTTTTT CCATGACATA TCTTCTCCTC TTTTTTCAAC
8480 CTCTTGGTGC ATTAATTTTC TTTTTAACAG GAAATATTTA GGGTAGATGT GTCTCCTCTT
8540 ACTCTGGAAA TGCGTGTGTG ATCCTCTTCC TTTTAATCAA CTTGGACCAT CGAGGGAAAA

8600 CTAATAAATT CTTGATCATT CAATTTGACT TTTCAAACCC AACCAAAAAA TGATTTTTGT
8660 AAAAAAATAA GTTCTTGAGA TCTGATTGAG AATTTGCTTG AGAAAGAGGA GGGGTTTTGG
8720 GATGTCCGTG ATTTAAGAAG GCTCTGTCCT TTACAGCTAG CAGCATCCAT AGTGTAGTGT
8780 GACTATGTCT AATATTACCT CTACTTTTAT TTGTGATTGA TGCTTAATGA TGAGAGGAAT
8840 ATTGCAAGTT AACAAATAAT TTACGCTAGA GTTTGTCTAG AATATTGAAA ATAATATATG
8900 AAGCTGTTCT TTCTCTTTGT TCAGTAAAAG GTACTAAATG TTTTCTCTTG TCTAATGTCT
8960 TCATCAGATC TGTGATTTTG GTCTTTCTCG TTTCAAAGCG AATACATTCC TTTCATCAAA
9020 GACTGCTGCC GGAACTGTAA ATTCTTAATT CGACTCTGTT GCCTTTATGT TCTGTTGATC
9080 TTTGACTTTT TTCTTGATGA TTGAATTGGG GAAATAAATG ATTTGGTGCT TATTGTGACA
9140 GCCGGAATGG ATGGCGCCTG AAGTTATTCG TGATGAACCA TCAAATGAGA AATCTGATGT
9200 ATACAGCTTT GGTGTCATTT TGTGGGAGCT GGCAACTCTT CAACAACCAT GGAATAAATT
9260 GAACCCACCA CAGGTTTGTA TTTGTTTGTT TGAAGTATTT TAATGTTAAG AGCATTTAGG
9320 TAAAAATGAT GATTTAAACA GTGCAATCTA GAGGTGAATA GTTTATATAT CAACAAAGGA
9380 CCAAAAGCTA TTTCTTGGGA TGTGAAAATG AGTCGGTCTC ATATGGCTCT GCCAGGGATT
9440 AGGGCACCAC AAGTCGGGAT GAGCCATTCC AGGAAGAGTC TTTTCAATTT GCTCTGCGTC
9500 ATCTACACTC TTCTTCATGT AGTTAATGTA GAAAGCTAGG CATTCATGAG CCATTGAATG
9560 GAAGAGTTCC AGGGGTACTA ATTTCTATTT GTGTGTAGGT TTTATTTTAC TTCTTGTGTA
9620 GTCTTTTAAT AGTTCATATT TTGTGCAACA TATATGTAAA CAGTTTGTCA AAATAATATT
9680 TAAGAAGAGA ACTATGGAAG TGGCGGCGGT GAGGTTGACG GCCAGCATAA AAGTAGTACG
9740 AGTATGATGA ATCATCTTAC TATAATTGTA ATGCCTGCCT ATCCAGACAT GTGCTATATG
9800 AGCACTGGAG TATGTGTTAA ATGAGCTCGG GGTTTCATCA CATCTCCCTC TTAGGTGAGA
9860 GCAAATATTT TTTATTAAAA AGAAAGTGAG AGCAAATATA AGCAAGGTTT CCCACACCAT
9920 ATACAGGTGT GACTAAAGAA ATTAGTCTGA CCACATTGAC GAATTAATGT GGTGCTCTAT
9980 AGGACAAGCT TGCCGTGTGT TAGAGGATGG GGAAGAAGAA GCTAATACAA GAGAGTAAGA
10040 TTCAAATAAG TTGTTGGAAT AGACTTGTTT GACGGGAAAG CAAATAGATT CAGCTCTGCT
10100 AGCATGTGGT GGAGTATCTG GGAAGGAGTG GAATGGAAGA TGTTAGGGGC ATTAGAGCTC
10160 AAGTTCAAAG TTTAATCTGA CTGTATTTTA TGGTCTCTTC CTGGTGTACA ATGGATGTAG
10220 TTGAGGATGC TGAAACCCTG TTACTGTTCA TAGATATTTT GTATTGATGG CCAGGGTTCA
10280 CTTTTTTTTT TCTCTGAGTG TAATCACAAC ACTTTCTTAG TGCTAGTGAC ATCAGTAATA
10340 GTTGCCAGTT CCCAAAAGAG AAGAAGGTGG ATGCTGAAAT CTAGAGATAT TGGTGTTAAA
10400 GAATGACAAA AAATCTCACA TCAGTGGTTA ATGAGATGGG TGGACTCCTT ATAAGGCTTG
10460 GACAATCTTC CTCCCTTGGA GCTAGCTTAT GGGGTGTGAG TTAGGCCTAA GACCTAATTT
10520 CACATGGTAT CAAAGCAGGT GGGGTGTTAA AGAATGACAA AAAGTCGGTT AATGAGATGG
10580 GTGGACTCCT TATAAGGCTT GGCAATCCTC CTCCTTTTGA GTTAGATTTT GGGGTGTGAG
10640 TTAGGTGTAA GACCTAATTT AACAATTGGC AACATATGTT GTAAAATGTG GTATTGTGGG
10700 ATAAAACTGG GAAGAAAGTG GGACTCATTG CAGTATTAGG ATTTAAAGTG AATTGTCGAA
10760 GTAAAGAAAA TCAGCGATAT GATTTATCTC ATTAAGTCTA TTGTAGGAAA AGTATGAACT
10820 CAACATCATG GGTTTCTATG TACTACAAAT AATAGACAAG AGAACTAAAA CAAAAATTTG
10880 GGAAGATATG GATAGTAGGA TAACCTTGGT CGAAGAGATC CCATGGGACT AGAGGCCATA
10940 GAAGGAAGGT AAATGGGAGT TGGAAGCTGA TGTAGATATT GTGGGAAGTA AATGACAAGT
11000 TGCATTATGA AAGCAATAAG AGTAGTCTTT GGTACATTTT AGAGAGGCTT AGGACTTGGG
11060 AGCTCAATTA GAGAAGCGTG TAAAGAGCTG TTAAAATAAA AAGACAGAAC AAATGGATTA
11120 TGAATATCAA AAGCCTTTAA AGAGTATTAT CAATATAAGA AGTCAGTTCC CTCATGTTCT
11180 TGTAGACCTC CATGTGCACC AGTCTATAGG TGCAGCAATA TGCAGGTTGA AGGCGATAAG

11240 AGGGCACAAG CACAAGAAGA AAAGTAGTTT CAGAAGATCA ATACAAGCTT ATGTAAGAAT
11300 AAACATAATA GAAGAAAAAA GATCCATATA GGGTACATTA GCTAGTTGGA TTAACTGTTG
11360 AGTTTAGCTT TTACACTTAT TTTGTGTCCA ATTTTGTGAG AAGTTCTCTT AATTGGGTAG
11420 AGACTCCTAT GTTCATGTAG AAATAAGTTT GATTCATGGA AGACCAAATG AGACAATACA
11480 ATATCAATGC CTCATGATTA TTAAAACTGT TTCTCGTTGA TATATAGTAC ATTCTAGCTT
11540 CTGCTACAAG TTTGATTTTT GAAAGATCAA ATAGAGGATC ATCCGTCAAC CCTCGTGATT
11600 ATTGAACTAT TCCCTTCTCT TTTCTTTTCT TTTCTTTTTT ATTATTGTGA ATGGCAGGAA
11660 ATGTGCTTTA ATGAATTTGA CTTTGTAGGT TATAGCAGCT GTCGGCTTTA ACAGGAAGAG
11720 GCTTGATATT CCAAGTGACT TGAATCCTCA AGTGGCGATT ATTATTGAGG CTTGCTGGGC
11780 TAAGTGTGTT TCTTTTACTT GTTCCCAAGT TTGCACCATT TAGCATCATG ATTGTTTTAA
11840 TGTGTTATCT TAAAGTTACT TTTGATGATG TTGCAGTGAG CCGTGGAAAC GCCCCTCCTT
11900 TTCCACTATC ATGGATATGC TGAGACCTCA TCTTAAATCT CCTCTACCTC CACCAGGTCA
11960 CACAGACATG CAGTTGCTCT CATGAATACC TGTGCTCTCT GCACATATTC TGCCGTCATT
12020 GCAAGCCATG TTTCTGGGAG GAAGAACATT TGGATTATGT CAGGTTTGTC TATCACCCAA
12080 ACTGTTTCTT CGTTACACTG AATTTTAAAG TTATTACCTT GGGTATGTCA CATCTTAGTT
12140 CAATTCTAGG TAAGTTAACT CTAGGTGACT GGTGCCAGCA GTAGTTGAAT AATTTGGTAG
12200 AATATTTGGA ATAACTTCTC TCGATGACAC TTTCTTGGGT TTTGGTGGAT CCTTTGGGAA
12260 TTGCTGGAGC CTGGAGCATC TGAACTGTTT GTGGATTGAG AAGGCATTAC TTTAGACTGA
12320 CATGCAGCCA GTATTCTGTG AGATGCCCAG CCTAACAAAC CTAGATATCG GAGTTTATTT
12380 TCGATAAAAA TGGGTCTAGC TCTTTGCTTT CTTGCAGTTC CAACGGCACA TGTTTTATGA
12440 CAGTACTTGT AAGCAAGTTT CTTCCCCTGT ATATGGCGGT GAAGTCTGAT GGATACATTG
12500 TATGTTTTTC AGGCTTGCAT GATGTACCTG TTCATATTAA TTCAGATTGG CTTTGGAGAA
12560 AACCAAGTTT TCTTCGCAAG CTCAAGCAGT GGCCACTGCC TCACAGAAGA TCCTTCGCAC
12620 TGGGCGTGTC CATTTCCTCT TTGAACCATT GTTTGAGCAA CTGATTTTGT TCAATTCCAA
12680 TTTTTTGTAA TTTCTGCCCA GGATTCTTGT ATAACTGTAT GTAAAAGCAT TATAATGGAA
12740 ATTTGGATCA TAAAGTACGT TTGTTTCATA TGA

>*LeEIN3*

-692 GTTTTTACTT TTAATGTCTT TGCACCTTCC TCTCTTCTTC TTACTTTCCA ATACACAACA
-632 AAAGTGAAAA CATCACTTCA ATACACCTAA CATTCTTCTC AAACCCCTTC TCTTTCTCTC
-572 TTTTTTCTTT TTTTTTGGTT CAAAATCAAA GTAGTATAGC CATAGATAAC AGCTTCAACA
-512 AGCTGTTTTC AAGAAAATCA TGTAAGTCAT TTTCTTGATT TTTTTTTGTT AAGTTTTTTT
-452 GAGTGTTGGT TTGTTGTGAA TTTTTGTGAA AATATGGTTC TTGAAGGGGT TGGTAGATCA
-392 GAGTATATGT GGGGATTAAA AGGGTGTTTT TTTTTTACAA ACAAAGAAGA TATAGATGGA
-332 ATTTTTAATA GTAATCAGTA GAGAAAATTA TGAAATTCTG TTAAAAGGGT GTTTTTCAGT
-272 AGAAAAAATT TGTTTTATGG CGTTGTTATA TTTATGTTTG TTTGTGTTTT TTTCTCCATG
-212 GAGTTATATT TATATGATGA CGTGTTATGT GTAGTATAAA GTGGGAGTAA TTTTTTATGA
-152 TTTGTTAAAG ATTATCTAGT TTTGAGGAAT TTGGACTGTT TTGTTTGATG ATGTTAGATT
-92 AAAGATCATT AATTTTGGAT TAAATTGGAG TTGATTTTAC TAAAAGTTGA GTTTATTTGT
-32 TGTATCTGAA TTGGTGTTGC AGAATTTGGT AA**ATG**GGGAT ATTTGAAGAT ATGGGGTTCT
29 CTGGAAATTT TGAGTTTCTA TCTGATTCTA TGGGATGTGG AGCTCAAGAA GTTGAGCATA
89 AGCCGGTTGG GTTGGAGGAG GATGATTATA GTGATGAGGA GATGGATGTG GAAGAGCTAG
149 AGAGGAGGAT GTGGAGGGAT CGAATGCTTT TGAGGCGTCT CAAAGAGAAA AACAAGAATA
209 AAGTGGTGGG GGATGGTGCG AAGCAGCGTC AGTCGCAGGA GCAGGCTCGT AGAAAGAAGA
269 TGTCGCGTGC ACAAGATGGT ATACTGAAGT ACATGCTGAA AATGATGGAG GTTTGTAATG

329 CTCAGGGTTT TGTTTATGGA ATTATCCCTG AGAAAGGGAA GCCTGTGACT GGTGCTTCGG
389 ACAATCTTCG TGCTTGGTGG AAGGAAAAGG TCAGATTTGA TCGAAATGGC CCTGCTGCTA
449 TTGCTAAGTA TCAGGCTGAT AATCAGATTC CTGGGAGAGT TGAGGAATCG AGTGTGATAG
509 TTTCCACTCC CCACACTTTA CAGGAGCTGC AGGATACAAC TCTAGGATCC CTTTTGTCTG
569 CTTTGATGCA GCACTGTGAT CCTCCACAGA GGCGGTTTCC GTTGGAGAAG GGGGTATCTC
629 CACCCTGGTG GCCCTCTGGT AAAGAGGAAT GGTGGGGTCA GTTGGGTCTG CCAAATGATC
689 AAGTTCAACC TCCATACAAG AAGCCTCATG ATCTGAAGAA GGCCTGGAAG GTTGGTGTTC
749 TGACGGCGGT AATCAAACAC ATCTCTCCCG ACATTGCTAA GATTCGCAAG CTTGTTCGAC
809 AGTCAAAGTG CTTGCAGGAT AAGATGACAG CTAAGGAGAG TGCTACTTGG CTTGCTATTA
869 TCAATCAAGA AGAGGCTTTG GCTCGTAAGC TGTATCCTGA CAGCTATCCA CAGGGATCTC
929 TAGCTGTTGG TAATGGTTCC TTTTTCATCA GCGATGCTAG CGATTACGAT GTGGAAGGAG
989 TGGATAACGA GAGAAACAAT GAAGTGGAAT GTAAACCCCA TGACATCAAT CTCCAAACTG
1049 GAATTATGTT ACCTAAAGAT AGGGTTTTGA TGCCAGGTTT AGCTCCAGTG AAAGGAGAAA
1109 TTATTGATTT AACTTCCGAT TTTATCCAGA AGAGGAAGGA ACCATGTTTT GAGGAGTCTG
1169 TTGATCAAAA GATATATACT TGTGAGTACC TTCACTGCCC ATACAGCAAT TATCAAGCTG
1129 GATTCCTTGA CAGGACTTCA AGAAACAACC ACCAAATGAG TTGTCCATTC CGGTTCAATT
1189 CTGCTCAAAC ACTTACTACA CCTAAGTATC AGATCAACTA TGAGCACAAC ACAGTTTTTC
1249 CTGCACAAAC TGCAACTTCT AAGCCAGCGG TCTCGTCAGT CACTGCTTCC TCTTCGATGA
1309 GTGCCTCGGG GCTTGGACTC CCTGAAGATG ATCAGAGGAT CATTTCTGAC CTCATAACAT
1369 CATACGACAA CAACTTTCAG CAAAATGGTA GCATCTGTTC CGGAATTTCT GAGATTCTAG
1429 TAAACCAAAG CCTGCCTCAG CAACAAACAG TTGAACTTCC CATGGATGGC AACATCAACC
1489 TAGGACATAT GGAGACCTCA GCTCAAGAAA CCAGCATGCC TGTTTATCGT TCAACAGAGT
1549 TTCAATATGA TCAATGCAAA ATGTCCTTTG ACGCCCCCTT CGGTGGAAAC ATAAATGATA
1609 TAACTGATTA CAGATTCGGT TCCCCGTTCA ACTTGGGAGG AAGCGACTAC GCCGTGGAAC
1669 AGCTGACAAA GCAGGATATA TCTACATGGT ACCTCTGAAC TAGTACTAGT ATTAAACTGT
1729 CTTATTTCCT ATATGAAGGC TTGATAGGTT GTATATGTTT AGATAAGTGA TCAACTCTCT
1789 GTCCTTTATA TATACAGGTA TTGTAGTTTC TTTGTGATGT AGTTGATGTT TCCATGGTTG
1849 TAAGTGCTAA ACACAATAAT TATCTTTATG CAATTATGTT TTAAACTCA

>SlERF-A1

-537 GATAACAATA AATGAAAGAA AAAACATATA CAATTATTCA AATTATCACC AAACCTATCC
-477 AACAACACAC TTCCACACCT CCCATAATAT TATCTCGATC AATCATATCA TAATTCTAAC
-417 GAAAAGCAAA AGAAAGACTT TAATGGAAGT CATGGTCCAT ATGTCTACGA CAAATAGTGA
-357 ATTACTAGAA AAATGCGGCT CAGTAATTTT ATCTTATTAG TATTATTCAA AATACCTAAT
-297 TTCCAATTTT CAAATACAAC ACGTGCTTAT TGAAACTCTC TATTTCCCTC TCAAAAACTT
-237 AGAACTTTTC TCCATTTAGA AGTTTCAAGG AAAGCTCAAA TTCCAAACAA ACAATATTTT
-177 AATCATTATT GTTATTAAAT TTTCCCACCC GTGCATGAAC CAAATTTCAT ATAAATATTT
-117 CCCTTTCCCC GCTTTAATCC AGGAAAAAAA AACTCATAAC TTCCTATTGT TTTTTCTTGA
-57 TTTGCTCTAT TTGTACTAAA TTCGCTTCGA TATAAAAATT CATAACCAAA ATTCAAA**ATG**
4 TATTCAAATT GTGAACTAGA AAATGATTTT TCAGTACTCG AATCAATTAG AAGATACTTA
64 CTTGAAGATT GGGAAGCTCC ATTAACGAGC TCTGAAAACT CAACATCCTC AGAGTTCAGC
124 CGGAGCAACA GCATTGAATC CAATATGTTT AGTAATTCAT TTGATTATAC ACCTGAAATT
184 TTTCAAAATG ATATTCTTAA TGAAGGATTT GGATTTGGAT TTGAATTCGA GACTTCTGAT
244 TTTATAATCC CTAAATTAGA GTCACAAATG TCAATCGAAT CACCTGAAAT GTGGAATTTA
304 CCGGAATTTG TGGCTCCATT AGAGACGGCG GCGGAGGTGA AAGTTGAAAC ACCGGTTGAG

364 ATGACAACTA CGACGACGAA GCCAAAGGCA AAGCATTATA GAGGTGTGAG AGTGAGGCCA
424 TGGGGGAAAT TCGCGGCGGA AATTAGAGAT CCGGCGAAAA ATGGAGCACG AGTTTGGCTC
484 GGTACATATG AGACGGCGGA GGATGCGGCG TTGGCTTACG ACAAGGCGGC TTTTCGCATG
544 CGGGGATCAC GTGCATTGCT GAATTTTCCG TTGAGGATTA ATTCCGGTGA ACCGGATCCT
604 GTTAGAGTTG GATCGAAGAG ATCGTCAATG TCGCCGGAGC ATTGTTCATC GGCGTCGTCG
664 ACGAAGAGGA GGAAGAAGGT TGCTCGTGGA ACAAAGCAAT AAGTCCTAAA AGTGGGCCCT
724 GTATAGTAAT AAAAAAAAAA TAGAATTATC CGACGGAAGT TGTTTTCTTA TAGAAAAAAG
784 TTATTATTTT GGGTGAAGTA ATGAATTTGT TTTTTCTTTC ATTTTTCGGC TGTGGTTGAT
844 GAAATCAACG AATCAAATAA TTATCCAAAA AATTAAGACA ATAATGTGAT AGTGATTATT
904 AAATAAATAA AAAGGTCAAA CTTACGTA
