# Supplementary material for: DNA Methylation Is Crucial for 1-Methylcyclopropene Delaying Postharvest Ripening and Senescence of Tomato Fruit
Source: Int J Mol Sci. 2024 Dec 28;26(1):168. doi: 10.3390/ijms26010168 (PMC11720368; doi:10.3390/ijms26010168)

SUPPLEMENTARY S2

Predicting the promoter and first exon regions of genes (-3000 bp to approximately 2000 bp) through CpG islands using CpG Island Online Prediction (MethPrimer 2.0)

SlACS1A (Solyc08g081550)

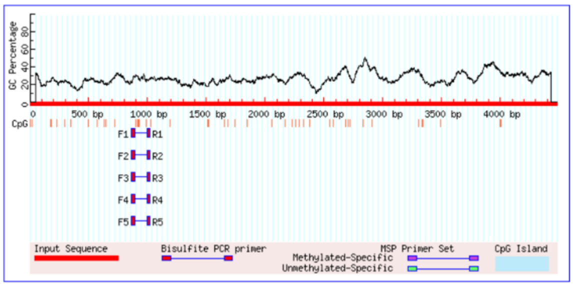

SlACS1B (Solyc08g081540)

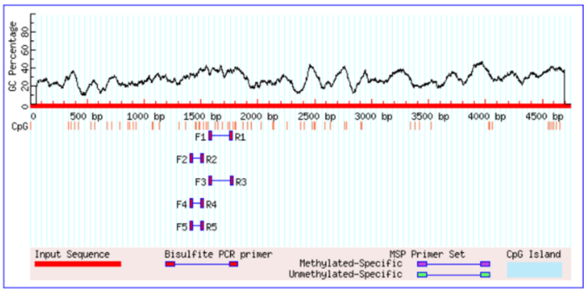

SlACS2 (Solyc01g095080)

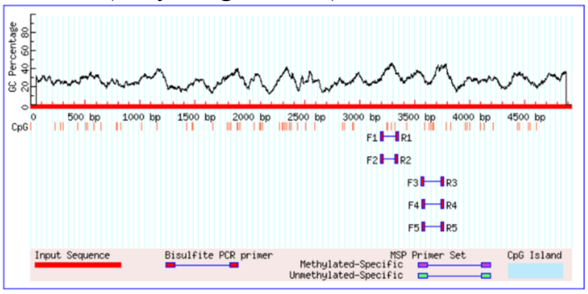

SlACS3 (Solyc02g091990)

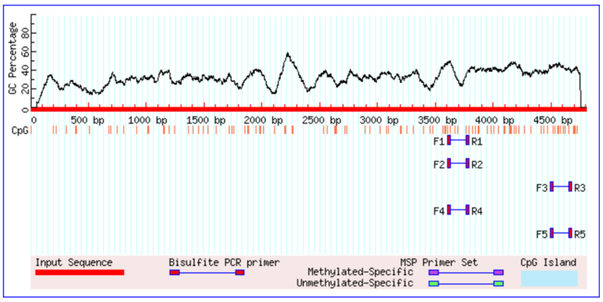

SlACS4 (Solyc05g050010)

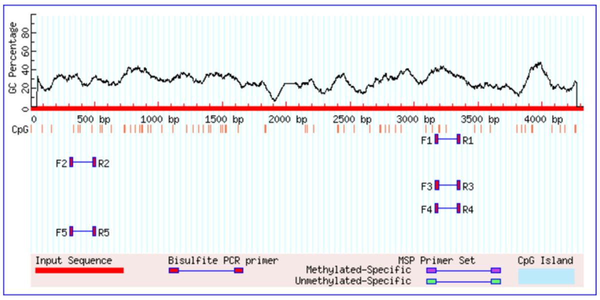

SlACS5 (Solyc04g077410)

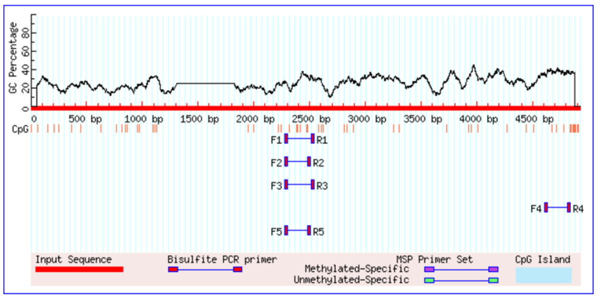

SlACS6 (Solyc08g008100)

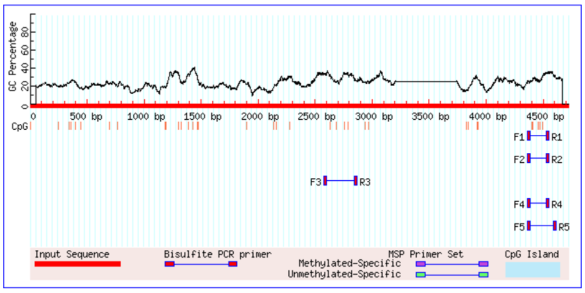

SlACS7 (Solyc02g063540)

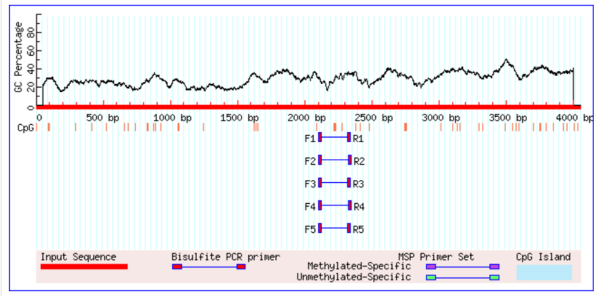

SlACS8 (Solyc03g043890)

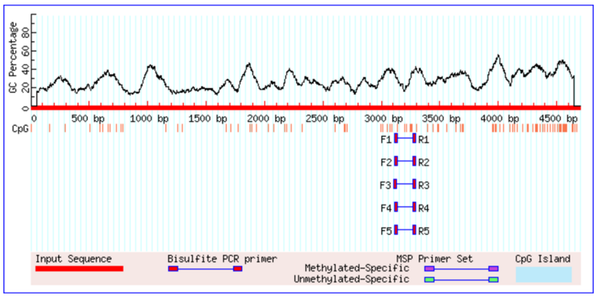

SlACS9 (Solyc07g026900)

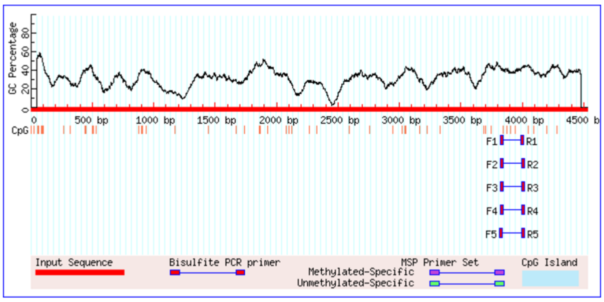

SlACS10 (Solyc12g008740)

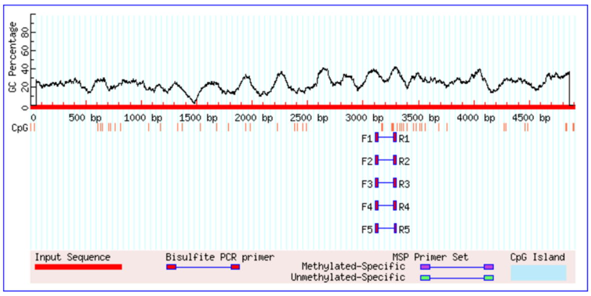

SlACS11 (Solyc03g007070)

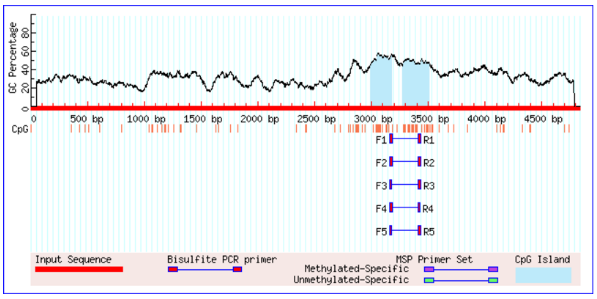

SlACS12 (Solyc08g079750)

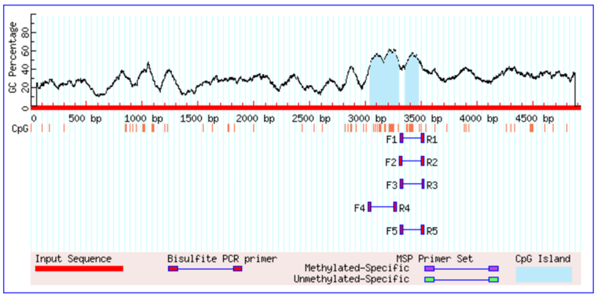

SlACS13 (Solyc12g056180)

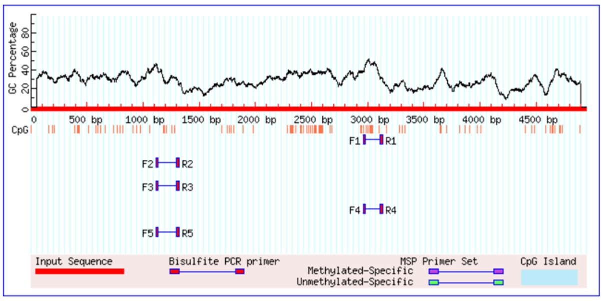

SlERF.C1 (Solyc05g051200)

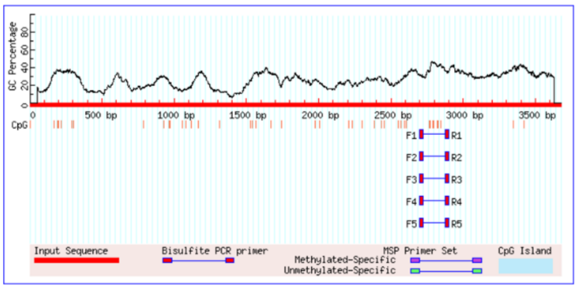

SlERF.D7 (Solyc03g118190)

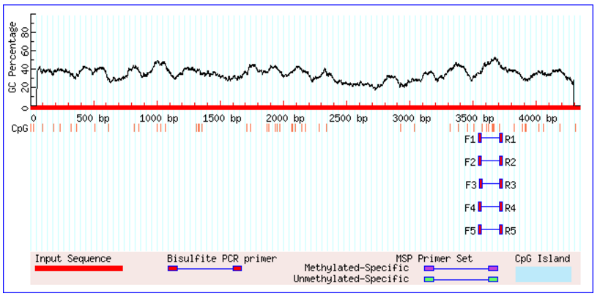

SlERF.A1 (Solyc08g078180)

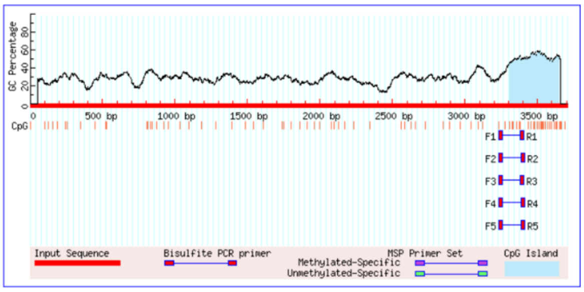

Supplement: Supplementary file 1 [file ijms-26-00168-s001.zip › Supplementary S2.pdf]
