# Supplementary material for: DNA Methylation Is Crucial for 1-Methylcyclopropene Delaying Postharvest Ripening and Senescence of Tomato Fruit
Source: Int J Mol Sci. 2024 Dec 28;26(1):168. doi: 10.3390/ijms26010168 (PMC11720368; doi:10.3390/ijms26010168)
Supplement: Supplementary file 1 [file ijms-26-00168-s001.zip › Table S1.pdf]

Table S1 forward and reverse primers used in qRT-PCR analyses

genes name	primer	Sequence (5’-3’)
SlACS10	Forward primer	GGTCGCAGAGGCAATCAAGC
	Reverse primer	GCCACAGCCCTCATTCTTATGC
LeCTR1	Forward primer	AAGAGAACCTGGCATCCG
	Reverse primer	TGAGGCAGACAGCGTTAC
LeEIN3	Forward primer	CATTGAAGCCGCCGTACAGA
	Reverse primer	TCAACCAATCTCACCTCGAAAGC
SlERF-A1	Forward primer	CACAGCCACTCAGAAGACCGTT
	Reverse primer	AGCACTTTCCCTACAGCCTTGG
