# Supplementary material for: DNA Methylation Is Crucial for 1-Methylcyclopropene Delaying Postharvest Ripening and Senescence of Tomato Fruit
Source: Int J Mol Sci. 2024 Dec 28;26(1):168. doi: 10.3390/ijms26010168 (PMC11720368; doi:10.3390/ijms26010168)
Supplement: Supplementary file 1 [file ijms-26-00168-s001.zip › Table S2.pdf]

Table S2 forward and reverse primers used in bisulfite sequencing PCR analyses

genes name	primer	Sequence (5'-3')
SlACS10	Forward primer	GGTTAGGTAGTTGATTGA(C/T)GTTATATT
	Reverse primer	CAAATACCTAAAATTACCCAATAATT
SlCTR1	Forward primer	GTATTTGATTTGGATTTGATGGATT
	Reverse primer	TACCAATACATCAATCACAAAATCC
LeEIN3	Forward primer	GTGGAGTTTAAGAAGTTGAGTATAAGT
	Reverse primer	CATCATTTTCAACATATACTTCAATAT
SlERF-A1	Forward primer	T(C/T)GGAATTTGTGGTTTTATTAGAGA
	Reverse primer	ATTACTTTATTCCAC(G/A)AACAACCTTC
